# An LCN2‐Dependent Positive‐Feedback Loop Between Gastric Cancer Cells and Tumor‐Associated‐Macrophages Mediates Lymphangiogenesis and Lymphatic Metastasis

**DOI:** 10.1002/advs.202508352

**Published:** 2025-08-30

**Authors:** Zhixin Huang, Ying Li, Yan Qian, Linying Ye, Tianhao Zhang, Yang Cheng, Jialin Wu, Peng Duan, Tiantian Zhang, Zihan Yu, Zeyu Zhao, Risheng Zhao, Zhi Liang, Ertao Zhai, Shirong Cai, Jianhui Chen

**Affiliations:** ^1^ Division of Gastrointestinal Surgery Center the First Affiliated Hospital of Sun Yat‐sen University Guangzhou Guangdong 510080 China; ^2^ Laboratory of Surgery the First Affiliated Hospital of Sun Yat‐sen University Guangzhou Guangdong 510080 China; ^3^ Guangdong Provincial Key Laboratory of Microbial Safety and Health State Key Laboratory of Applied Microbiology Southern China Institute of Microbiology Guangdong Academy of Sciences Guangzhou Guangdong 510070 China; ^4^ Department of General Surgery Guangxi Hospital Division of The First Affiliated Hospital Sun Yat‐sen University Nanning Guangxi 530000 China

**Keywords:** gastric cancer, lipocalin‐2, lymph node metastasis, lymphangiogenesis, tumor microenvironment

## Abstract

Lymph node (LN) metastasis is a major determinant of poor prognosis in patients with gastric cancer (GC). Tumor‐associated macrophages (TAMs) play a crucial role in promoting tumor metastasis and progression; however, the underlying mechanisms through which TAMs induce LN metastasis in GC remain poorly understood. This study demonstrates that low lipocalin‐2 (LCN2) expression is associated with increased LN metastasis and shorter survival in GC. Functionally, LCN2 silencing significantly increases M2‐type TAM infiltration, lymphangiogenesis, and LN metastasis. Mechanistically, LCN2 downregulates the NF‐κB pathway‐mediated CCL5 expression by interacting with Annexin A1, which inhibits K63‐ and M1‐linked ubiquitination of NEMO. Furthermore, LCN2‐regulated CCL5 recruits and repolarizes TAMs through the CCR5/PI3K/AKT/GSK3β axis, which subsequently promotes lymphangiogenesis and LN metastasis via vascular endothelial growth factor C (VEGFC) secretion. Additionally, interleukin‐10 (IL‐10) derived from M2‐type TAMs suppresses IκBζ and its target gene, *LCN2*, in GC cells by promoting IκBζ degradation, thereby establishing an IL‐10/IκBζ/LCN2 positive‐feedback loop that sustains LCN2 suppression. These findings suggest that reduced LCN2 expression drives a positive feedback loop between tumor cells and TAMs that continuously enhances lymphangiogenesis and LN metastasis in GC. Therefore, targeting these related pathways may represent a promising therapeutic strategy for GC patients and LN metastasis.

## Graphical abstract

In this study, the expression and function of LCN2 in GC is aimed to investigate and elucidate the mechanisms by which the LCN2‐dependent positive‐feedback loop between GC cells and TAMs drives lymphangiogenesis and lymphatic metastasis. These findings suggest that LCN2 may serve as a potential prognostic biomarker and therapeutic target in GC.

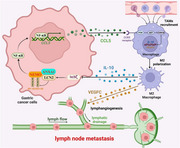

## 1 Introduction

Among malignancies of the digestive tract, gastric cancer (GC) has one of the highest incidence and mortality rates, with approximately one million new cases and 76 9000 deaths reported worldwide in 2020.^[^ ^1^, ^2^ ^]^ Lymph node (LN) metastasis is the predominant mode of GC metastasis, occurring not only in advanced stages but also in the early stages of the disease.^[^ ^3^, ^4^, ^5^ ^]^ LN metastasis significantly worsens the prognosis of patients with GC following curative gastrectomy, reportedly reducing the 5‐year survival rate from 95% to 33.3%.^[^ ^6^, ^7^ ^]^ However, effective and reliable targets for identifying or controlling LN metastasis remain lacking. Therefore, elucidating the mechanisms underlying lymphatic metastasis in GC and identifying potential therapeutic targets for patients with LN metastasis are of critical clinical importance.

Lymphangiogenesis, the formation of new lymphatic vessels, is a crucial rate‐limiting step in LN metastasis across various solid tumors.^[^ ^8^, ^9^ ^]^ These newly formed lymphatic vessels not only facilitate tumor cell recruitment and dissemination but also influence tumor stem cell survival, immune responses, and inflammatory processes.^[^ ^10^, ^11^ ^]^ Vascular endothelial growth factor C (VEGFC), secreted by tumor cells, immune cells, and other components of the tumor microenvironment (TME), is a key driver of lymphangiogenesis.^[^ ^12^, ^13^ ^]^ Furthermore, VEGFC promotes the proliferation and migration of lymphatic endothelial cells by binding to its receptor Vascular endothelial growth factor receptor 3 (VEGFR3) on these cells, leading to lymphangiogenesis in tumors.^[^ ^14^, ^15^ ^]^ Notably, recent studies have highlighted the role of various tumor‐associated immune cells—such as tumor‐associated neutrophils, cancer‐associated fibroblasts, mast cells, and basophils—in promoting lymphangiogenesis by regulating VEGFC/VEGFR3 signaling.^[^ ^16^, ^17^, ^18^ ^]^ Among these, tumor‐associated macrophages (TAMs) are the most abundant immune cells in the TME. Depending on the signals they receive, TAMs can polarize into either a pro‐tumorigenic M2 phenotype or an anti‐tumorigenic M1 phenotype.^[^ ^19^ ^]^ M2‐type TAMs promote tumor metastasis by directly regulating epithelial‐mesenchymal transition (EMT), remodeling the extracellular matrix (ECM), inducing tumor angiogenesis and pre‐metastatic niche formation, and mediating immune suppression.^[^ ^20^, ^21^ ^]^ Additionally, infiltrating TAMs have been shown to facilitate lymphangiogenesis and LN metastasis by upregulating VEGFC.^[^ ^22^, ^23^ ^]^ However, the specific roles and regulatory mechanisms of infiltrating TAMs in lymphangiogenesis and LN metastasis in GC remain largely unexplored.

Lipocalin‐2 (LCN2), a member of the adipokine family, has been implicated in the development and progression of various tumors.^[^ ^24^, ^25^ ^]^ In colorectal cancer, LCN2 inhibits metastasis by attenuating the NF‐κB‐mediated activation of Snail and EMT.^[^ ^26^ ^]^ In non‐small cell lung cancer, LCN2 reduces tumor chemosensitivity to almonertinib via the LCN2‐matrix metalloproteinase 9 pathway.^[^ ^27^ ^]^ Similarly, studies have suggested a link between LCN2 and GC progression; however, while some reports indicate that LCN2 promotes GC progression, others present conflicting findings.^[^ ^28^, ^29^, ^30^ ^]^ Furthermore, our previous studies have shown that LCN2 is negatively associated with GC progression.^[^ ^31^, ^32^ ^]^ These conflicting findings highlight the need for further investigation of the precise role of LCN2 in GC and the underlying mechanisms. More importantly, the function and underlying mechanisms by which LCN2 influences lymphangiogenesis, LN metastasis, and TME remodeling in GC remain largely unexplored.

This study aimed to investigate the expression and function of LCN2 in GC and elucidate the mechanisms by which the LCN2‐dependent positive feedback loop between GC cells and TAMs drives lymphangiogenesis and lymphatic metastasis. Our findings suggest that LCN2 may serve as a potential prognostic biomarker and therapeutic target in GC.

## 2 Results

### 2.1 Lowered LCN2 is Associated With Increased Lymph‐Node Metastasis and Shorter Survival in GC

**Figure 1:**
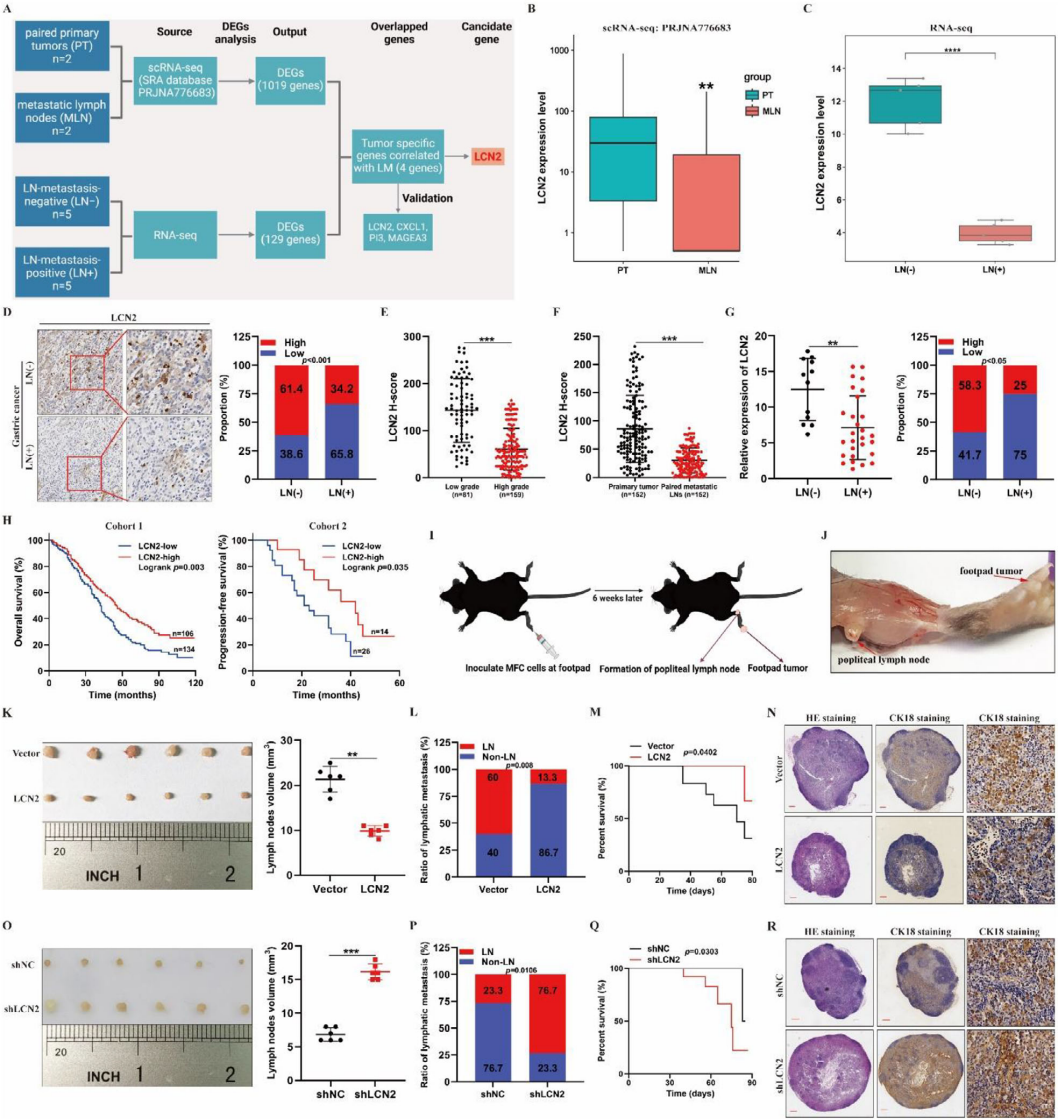
Lowered‌ LCN2 is associated with increased lymph‐node metastasis and shorter survival in GC. **A)** Flowchart depicting the identification of tumor‐specific genes correlated with lymph node metastasis in GC. **B)** *LCN2* expression levels in primary tumors (PT) and metastatic lymph nodes (*MLN*) of gastric cancer based on our previous single‐cell RNA sequencing data. **C** *LCN2* expression levels in five LN‐metastasis‐negative (*LN−*) and five LN‐metastasis‐positive (*LN+*) primary gastric cancer tissues based on RNA sequencing data. **D)** Representative IHC images (left) and quantification (right) of *LCN2* expression in paraffin‐embedded sections of primary gastric cancer tissues with (*n* = 152) or without (*n* = 88) LN metastasis. **E)** Correlation between *LCN2* expression and pathological grade in primary gastric cancer tissues as assessed by IHC (*n* = 240). **F)** IHC analysis of *LCN2* expression in primary gastric cancer tissues compared with paired metastatic LN tissues (*n* = 152). **G)** RT‐qPCR analysis (left) and quantification (right) of *LCN2* mRNA expression in freshly collected gastric cancer tissues with (*n* = 28) or without (*n* = 12) LN metastasis. **H)** Kaplan‐Meier survival analysis of OS (left) and PFS (right) in GC patients with low versus high *LCN2* expression. **I,J)** Schematic illustration I) and representative images J) of the *615‐line* mouse model of popliteal LN metastasis. **K,O)** Representative images (left) and volume measurements (right) of enucleated popliteal lymph nodes in the indicated groups (*n* = 15 per group). **L,P)** Proportion of metastatic and non‐metastatic popliteal lymph nodes in the indicated groups. **M,Q)** Kaplan‐Meier survival analysis of mice in the indicated groups. **N,R)** Representative images of HE and CK‐18 IHC staining of popliteal lymph nodes from the *LCN2*‐overexpression group L) and *LCN2*‐knockdown group P). Data are expressed as mean ± SD of biological replicate experiments. ** indicates *p *< 0.01, and *** indicates *p *< 0.001.

To identify potential genes associated with LN metastasis in GC, we performed transcriptome sequencing on five LN‐metastasis‐negative (LN−) and five LN‐metastasis‐positive (LN+) primary GC tissue samples. Patient characteristics are summarized in Table S1 (Supporting Information). Additionally, we analyzed the single‐cell RNA sequencing data from our previous study (SRA database PRJNA776683) on primary tumors (PTs) and paired metastatic lymph nodes (MLNs) in GC. As illustrated in the flowchart (**Figure** **1**A), we cross‐referenced datasets comparing PTs with MLNs and LN− with LN+ primary GC tissues. Through this analysis, we preliminarily identified four genes—*LCN2, CXCL1, PI3*, and *MAGEA3*—that correlated with LN metastasis by overlapping the two datasets (Tables S7 and S8, Supporting Information). Further validation revealed that *LCN2*, but not the other genes, consistently influenced GC progression and LN metastasis across multiple analyses (Figure 1B,C; Figure S1A–G, Supporting Information). Consequently, we focused on *LCN2* for further investigation. *LCN2* expression was significantly downregulated in metastatic GC cells in both sequencing datasets (Figure 1B,C). To further validate these findings, we examined *LCN2* protein expression in paraffin‐embedded GC tissue samples from 240 patients. The results indicated that LCN2 expression was significantly lower in LN+ GC tissues than in LN–GC tissues and was strongly correlated with tumor pathological grade (Figure 1D,E; Table S2, Supporting Information). Additionally, immunohistochemistry (IHC) revealed lower *LCN2* expression in metastatic tumor cells within MLNs than in the paired PTs (Figure 1F), suggesting that GC cells with lower *LCN2* expression are more likely to metastasize to LNs. To further confirm these findings, we examined *LCN2* mRNA expression in GC tissue from an independent cohort of 40 patients. *LCN2* mRNA expression was also significantly downregulated in the GC samples with LN metastasis (Figure 1G). More importantly, low *LCN2* expression was associated with poorer overall survival (OS) and progression‐free survival (PFS) in patients with GC (Figure 1H). We also analyzed *LCN2* mRNA expression in The Cancer Genome Atlas (TCGA) database, which showed a negative correlation between *LCN2* expression and both the pathological grade and clinical stage of tumors in patients with GC (Figure S1A, Supporting Information). Furthermore, data analysis from the TCGA and Kaplan‐Meier plotter databases confirmed that lower *LCN2* expression was associated with a worse prognosis in patients with GC (Figure S1B, Supporting Information). Overall, these findings suggest that *LCN2* plays an inhibitory role in the regulation of LN metastasis in GC.

To further investigate the role of *LCN2* in LN metastasis in GC, we established an in vivo popliteal lymphatic metastasis model using mouse forestomach carcinoma (MFC) cells with stable *LCN2* overexpression or silencing (Figure 1I,J). The results showed that *LCN2* overexpression reduced the volume of popliteal LNs (Figure 1K), whereas *LCN2* silencing had the opposite effect (Figure 1O). Meanwhile, we found that the differential expression of LCN2 in MFC cells has a minimal impact on the growth of primary tumors (Figure S1J,K, Supporting Information). Further, IHC staining demonstrated that *LCN2* overexpression significantly inhibited LN metastasis (Figure 1L,N; Figure S1H, Supporting Information), whereas *LCN2* silencing enhanced metastasis from the PT to the popliteal LNs (Figure 1P,R; Figure S1I, Supporting Information). Moreover, survival analysis revealed that mice with *LCN2*‐silenced tumors had shorter survival times, whereas those with *LCN2*‐overexpressing tumors had longer survival times than mice in the control group (Figure 1M,Q). Collectively, these findings demonstrate that *LCN2* silencing promotes LN metastasis of GC in vivo.

### 2.2 TAMs are Integral for LCN2‐Mediated Inhibition of Lymph‐Node Metastasis in GC

**Figure 2:**
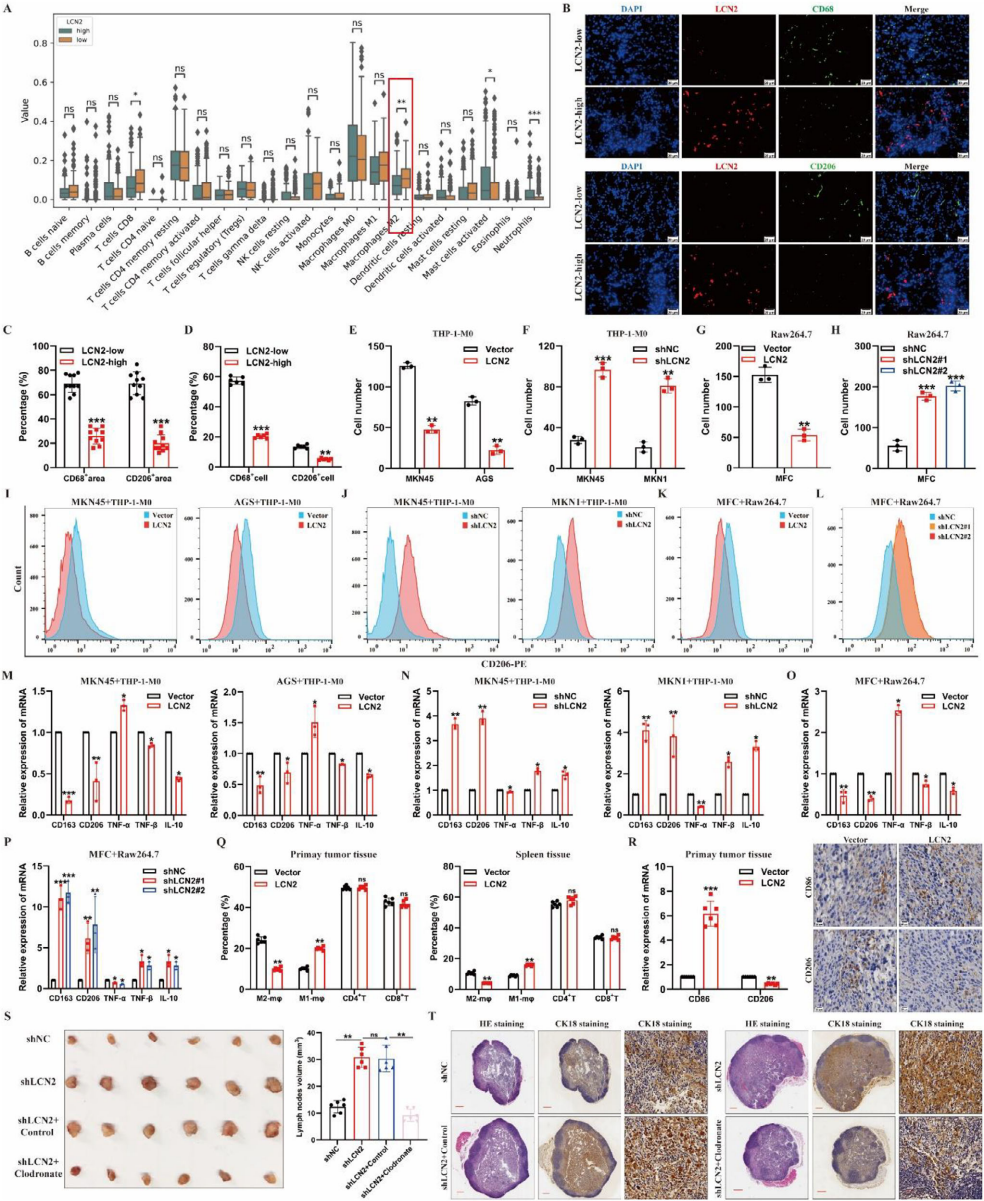
Tumor‐associated macrophages are integral for LCN2‐mediated inhibition of lymph‐node metastasis in GC. **A)** Estimated intra‐tumoral levels of 22 immune cell types in gastric cancer tissues with high or low *LCN2* expression from TCGA‐STAD, assessed via CIBERSORT. **B,C)** Representative immunofluorescence staining images B) for CD68 and CD206, along with quantification C) of CD68^+^ and CD206^+^ cells infiltrated in paraffin‐embedded sections of gastric cancer tissues with different *LCN2* expression levels. **D)** Proportions of CD45^+^CD68^+^ macrophages and CD206^+^ TAMs in fresh, surgically excised human GC tissues with varying *LCN2* expression levels. **E–H)** Quantitative transwell assay results for THP‐1‐derived M0 macrophages E,F) and Raw264.7 cells G,H), assessing the chemotactic response to conditioned medium collected from the indicated GC cells. **I–L)** Proportions of CD206^+^ cells in THP‐1‐derived M0 macrophages I,J) and Raw264.7 cells K,L) following co‐culture with the indicated GC cells. **M–P)** Expression levels of M1‐like and M2‐like macrophage biomarkers in THP‐1‐derived M0 macrophages M,N) and Raw264.7 cells O,P) after co‐culture with the indicated GC cells. **Q)** Flow cytometry analysis of CD45^+^F4/80^+^CD206^+^ cells, CD45^+^F4/80^+^CD86^+^ cells, CD45^+^CD3^+^CD4^+^ cells, and CD45^+^CD3^+^CD8^+^ cells in tumor and spleen tissues of mice from the indicated groups. **R)** RT‐qPCR and IHC analyses of CD86 and CD206 expression in primary tumor tissues of mice from the indicated groups. **S)** Representative images (left) and volume measurements (right) of enucleated popliteal LNs in the indicated groups (*n* = 15 per group). **T)** Representative HE and CK‐18 IHC staining images of popliteal lymph nodes from the indicated groups. Data are expressed as mean ± SD of biological replicate experiments. ** indicates *p *< 0.01, *** indicates *p *< 0.001 and ns indicates no significance.

It is well established that immune cells within the TME are involved in various biological processes of tumor progression. To determine whether *LCN2* downregulation promotes lymphatic metastasis in GC by modulating the TME, we first analyzed the relationship between *LCN2* expression and the abundance of various immune cells using the TCGA database. The results revealed that tumors with low *LCN2* expression were enriched in M2‐type TAMs (**Figure** **2**A), leading us to hypothesize that M2‐type TAMs might be involved in *LCN2*‐dependent lymphatic metastasis in GC. Further analysis of TCGA‐STAD samples showed that higher *LCN2* expression was associated with reduced tumor infiltration of M2‐type TAMs and increased tumor infiltration of M1‐type TAMs (Figure S2A,B, Supporting Information). Similarly, correlation analysis revealed a positive correlation between *LCN2* expression and M1‐type TAM markers expression (*NOS2*, *TNF‐α*) and a negative correlation with M2‐type TAM markers expression (*CD163*, *CD206*, *CD301*) (Figure S2C, Supporting Information). Next, we assessed TAM and M2‐type TAM infiltration in human GC tissues with different *LCN2* expression levels using flow cytometry and immunofluorescence. The proportions of TAMs (*CD45⁺CD68⁺*) and M2‐type TAMs (*CD45⁺CD68⁺CD206⁺*) were both negatively correlated with *LCN2* expression (Figure 2B–D; Figure S2D–F, Supporting Information). To evaluate whether *LCN2* directly regulates macrophage recruitment and polarization in vitro, we constructed a GC cell–macrophage co‐culture model using transwell chambers (Figure S2G, Supporting Information). First, we constructed stable LCN2‐overexpressing and silencing GC cells based on the expression level of LCN2 in common GC cells. Conditioned medium (CM) from *LCN2*‐overexpressing GC cells significantly reduced the recruitment and M2‐type polarization of Human Monocytic Leukemia Cell Line 1 (THP‐1)‐derived M0 macrophages and RAW264.7 cells (Figure 2E–O; Figure S2H,J, Supporting Information). Conversely, CM from *LCN2*‐silenced GC cells promoted the recruitment and M2‐type polarization of THP‐1‐derived M0 macrophages and RAW264.7 cells (Figure 2F–P; Figure S2I,K, Supporting Information). These results remained consistent in different GC cell lines. Moreover, in the popliteal lymphatic metastasis mouse model, *LCN2* overexpression decreased the proportion of M2‐type TAMs while increasing M1‐type TAMs in PTs and spleen tissues (Figure 2Q; Figure S2L, Supporting Information). Correspondingly, *LCN2* overexpression resulted in reduced *CD206* expression and increased *CD86* expression in footpad PT tissue (Figure 2R). However, analysis of the proportions of CD4^+^ T‐cells and CD8^+^ T‐cells in footpad PT and spleen tissues revealed no significant changes with *LCN2* overexpression or silencing (Figure 2Q; Figure S2L, Supporting Information). To further determine the critical role of macrophages in *LCN2*‐mediated inhibition of lymphatic metastasis in GC, we depleted macrophages in mice using clodronate liposomes and then constructed the popliteal lymphatic metastasis model. We found that macrophage depletion prevented the promotion of LN metastasis induced by *LCN2* silencing (Figure 2S,T; Figure S2M, Supporting Information). Taken together, these results demonstrate that TAMs play a crucial role in the enhanced lymphatic metastasis observed following *LCN2* silencing in GC.

### 2.3 CCL5 is Essential for LCN2‐Mediated Inhibition of Macrophage Activation and Lymph‐Node Metastasis in GC

**Figure 3:**
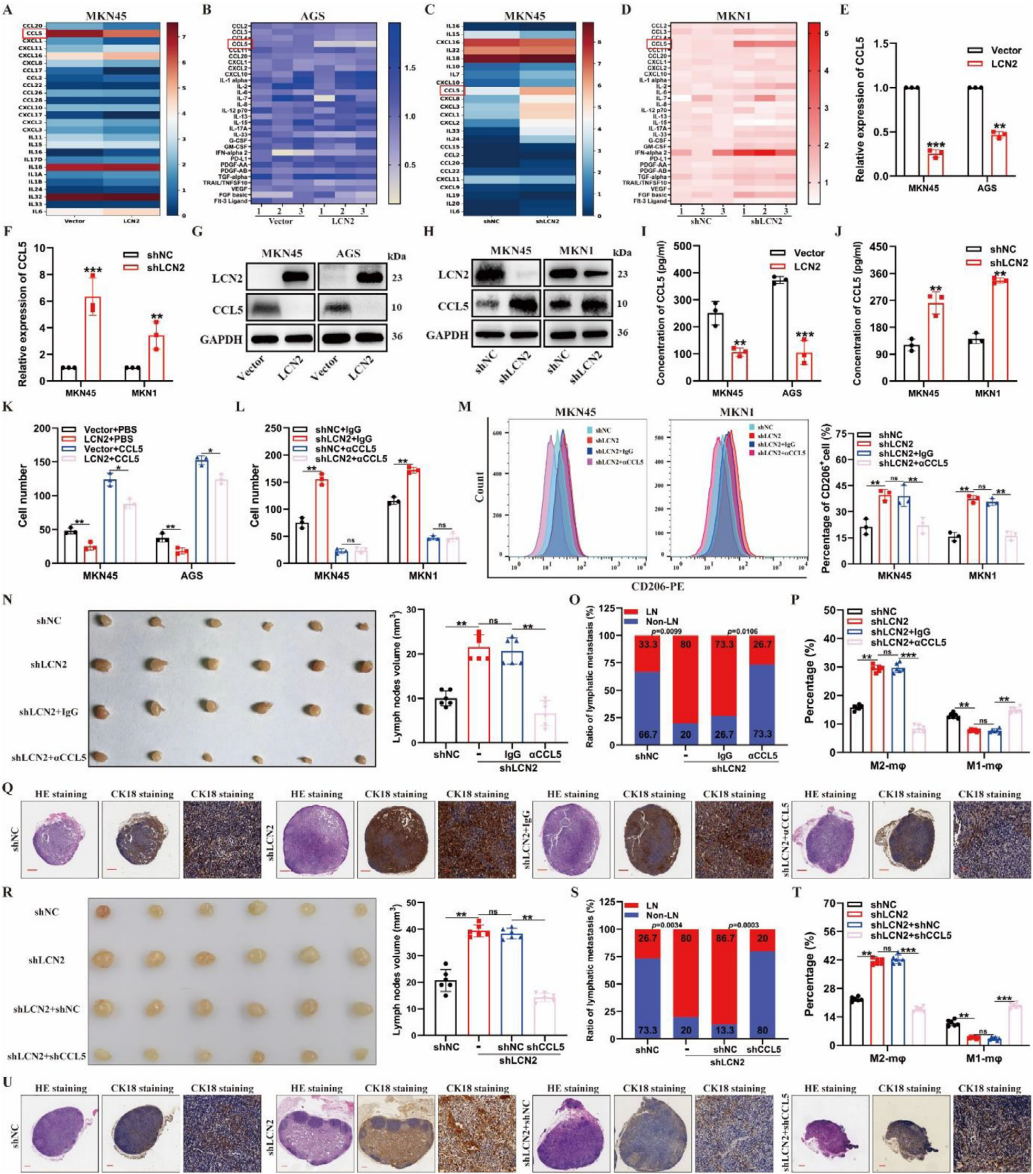
CCL5 is essential for LCN2‐mediated inhibition of macrophage activation and lymph‐node metastasis in GC. **A,C)** Heatmaps showing differentially expressed cytokines and chemokines regulated by *LCN2*, based on transcriptome sequencing analysis. Rows represent cytokine and chemokine names, and columns represent treatment conditions. *CCL5* is highlighted. **B,D)** Luminex assay analysis of 46 cytokines and chemokines in conditioned medium from the indicated GC cells. **E–J)** RT‐qPCR (E, F), western blot G,H), and ELISA I,J) analyses of *CCL5* expression in *LCN2*‐overexpressing (LCN2), *LCN2*‐silenced (shLCN2), and control GC cells. **K,L)** Quantitative transwell assay results for THP‐1‐derived M0 macrophages following the indicated treatments. **M)** Representative flow cytometry images (left) and quantification (right) of CD206^+^ cells in THP‐1‐derived M0 macrophages under the indicated conditions. **N,R)** Representative images (left) and volume measurements (right) of enucleated popliteal LNs in the indicated mouse groups. **O,S)** Proportion of metastatic and non‐metastatic popliteal lymph nodes in the indicated groups. **P,T)** Flow cytometry analysis of CD45^+^F4/80^+^CD206^+^ and CD45^+^F4/80^+^CD86^+^ cells in tumor tissues from the indicated mouse groups. **Q,U)** Representative HE and CK‐18 IHC staining images of popliteal lymph nodes from the indicated groups. Data are expressed as mean ± SD of biological replicate experiments. ** indicates *p *< 0.01, *** indicates *p *< 0.001, and ns indicates no significance.

Next, we attempted to clarify the mechanism by which *LCN2* silencing in GC cells promotes M2‐type TAM infiltration. Chemokines and cytokines play a central role in intercellular communication that drives tumor metastasis, progression, and therapeutic resistance.^[^ ^33^ ^]^ To identify the chemokines and cytokines involved in *LCN2*‐induced TAM recruitment and M2‐type polarization, we performed RNA sequencing and Luminex assays. The results revealed significant changes in chemokine expression following *LCN2* overexpression or silencing, with *CCL5* expression and secretion showing the most pronounced changes in both expression levels and secretion (**Figure** **3**A–D; Tables S6,S9,S10, Supporting Information). Specifically, *CCL5* expression and secretion were significantly decreased in *LCN2*‐overexpressing GC cells and increased in *LCN2*‐silenced cells (Figure 3A–D; Table S6, S9 and S10, Supporting Information). We further validated these findings using quantitative reverse transcription polymerase chain reaction (RT‐qPCR), western blotting (WB), and enzyme‐linked immunosorbent assay (ELISA) (Figure 3E–J; Figure S3A, Supporting Information). Collectively, these results indicate that *LCN2* silencing can enhance *CCL5* expression and secretion. To determine whether increased *CCL5* secretion is responsible for macrophage activation and LN metastasis induced by *LCN2* silencing, we first analyzed the relationship between *CCL5* expression and macrophage/M2‐type TAM infiltration in TCGA‐STAD samples. The analysis revealed a positive correlation between *CCL5* expression and the proportion of macrophages and M2‐type TAMs (Figure S3B,C, Supporting Information). Next, we examined the effect of *CCL5* on *LCN2*‐mediated recruitment and M2‐type polarization of macrophages in vitro. Treatment with CM from *LCN2*‐silenced GC cells significantly increased the recruitment and M2‐type polarization of THP‐1‐derived M0 macrophages (Figure 3K–M; Figure S3D,E, Supporting Information). However, this effect was partially mitigated by the addition of a *CCL5*‐neutralizing antibody (Figure 3K–M; Figure S3D,E, Supporting Information), suggesting that *CCL5* inhibition abrogates the promoting effect of *LCN2* silencing on macrophage activation. Furthermore, we found that *CCL5* promoted the recruitment and M2‐type polarization of THP‐1‐derived M0 macrophages in a concentration‐dependent manner (Figure S3F,G, Supporting Information). To further investigate the influence of *CCL5* on *LCN2*‐mediated lymphatic metastasis, we assessed the effects of *CCL5* inhibition in vivo using both a *CCL5*‐neutralizing antibody and RNA interference (shRNA). Treatment with the *CCL5*‐neutralizing antibody significantly reduced MFC cell metastasis to LNs (Figure 3N–Q). Additionally, *CCL5* inhibition partially blocked the increase in lymphatic metastasis and M2‐type TAM infiltration while preventing the decrease in M1‐type TAMs induced by *LCN2* silencing (Figure 3N–Q; Figure S3H,J, Supporting Information). Deletion of *CCL5* in *LCN2*‐silenced MFC cells yielded results consistent with those obtained using the *CCL5*‐neutralizing antibody (Figure 3R–U; Figure S3I,K, Supporting Information). Overall, these findings demonstrate that tumor cell‐derived *CCL5* is a key mediator of macrophage activation and lymphatic metastasis induced by *LCN2* silencing in GC.

### 2.4 LCN2 downregulates CCL5 expression by inhibiting the activation of the NF‐κB pathway via Annexin A1 (ANXA1)‐dependent poly‐ubiquitination of NF‐κB essential modulator (NEMO)

**Figure 4:**
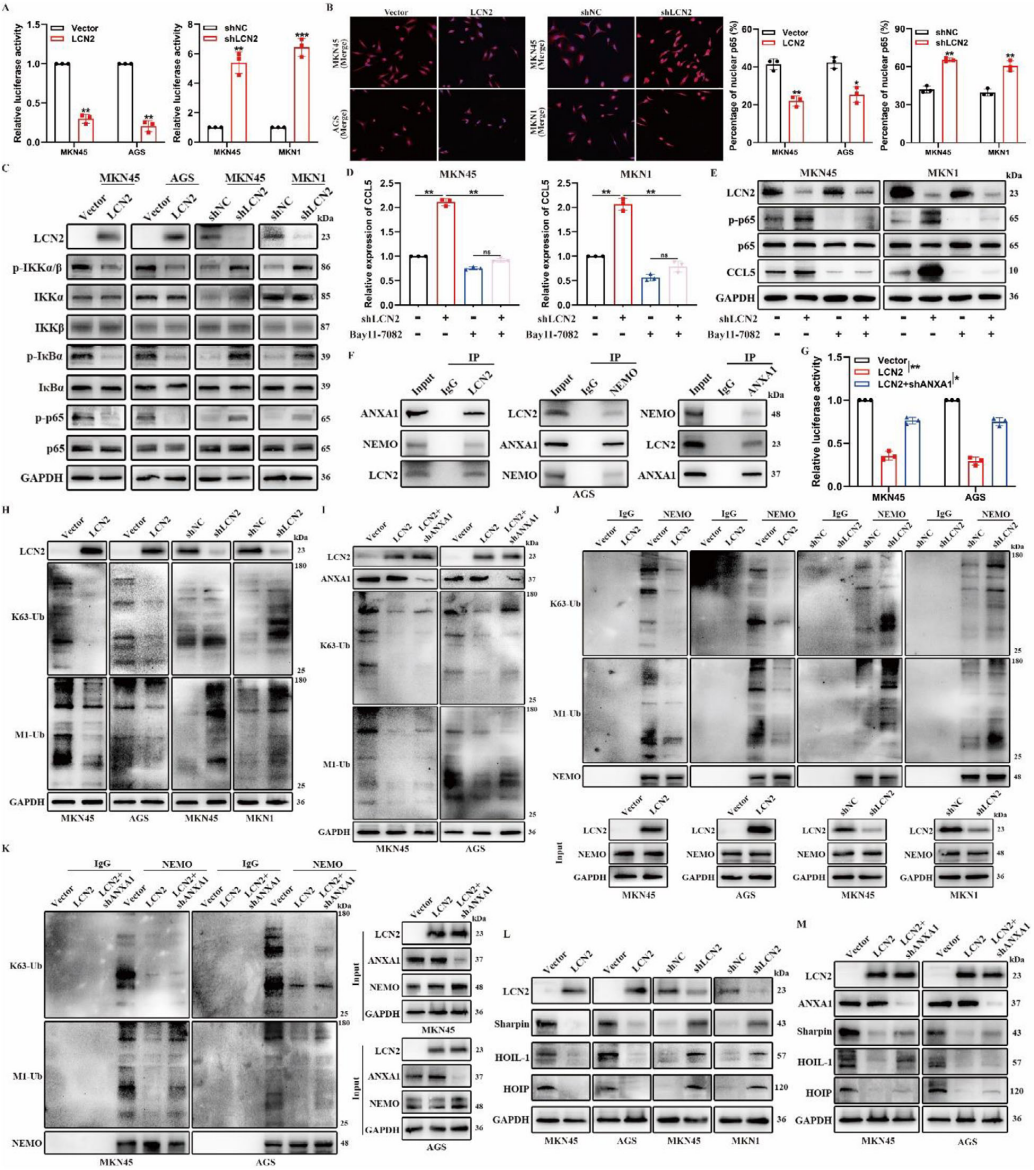
LCN2 downregulates CCL5 expression by inhibiting the activation of the NF‐κB pathway via ANXA1‐dependent poly‐ubiquitination of NEMO. **A)** NF‐κB luciferase reporter activity in the indicated GC cells with different LCN2 expression levels. **B)** Representative immunofluorescence staining images (left) and quantification (right) of the subcellular localization of NF‐κB/p65 in the indicated GC cells with different LCN2 expression levels. Scale bar, 50 µm. **C)** Western blot analysis of NF‐κB pathway‐related protein expression in the indicated GC cells with different LCN2 expression levels. **D,E)** RT‐qPCR D) and western blot E) analyses of the expression of CCL5 in LCN2‐silenced and control GC cells treated with or without the indicated dose of the NF‐κB pathway inhibitor Bay11‐7082. **F)** The lysates of AGS cells transfected with an LCN2‐overexpression plasmid were immunoprecipitated with antibodies against LCN2, ANXA1, and NEMO. **G)** NF‐κB luciferase reporter activity in the indicated GC cells with the corresponding treatment. **H,I)** Western blot analysis of K63‐Ub and M1‐Ub expression in the indicated GC cells with the corresponding treatment. **J,K)** Western blot analysis of K63‐Ub and M1‐Ub expression in the lysates of the indicated GC cells with the corresponding treatment, immunoprecipitated with antibodies against NEMO. **L,M)** Western blot analysis of the expression of LUBAC‐related proteins, Sharpin, HOIP, and HOIL‐1 in the indicated GC cells with the corresponding treatment. Data are expressed as the mean ± SD of biological replicate experiments. * indicates *p *< 0.05, ** indicates *p *< 0.01, and *** indicates *p *< 0.001.

We subsequently sought to elucidate the precise mechanism by which *LCN2* inhibits *CCL5* expression. *CCL5* is a downstream target gene of the NF‐κB pathway,^[^ ^34^, ^35^ ^]^ and its promoter region contains potential binding sites for NF‐κB/p65. Furthermore, previous studies have suggested that *LCN2* acts as an upstream regulator of the NF‐κB pathway and can attenuate its activation.^[^ ^26^ ^]^ Based on these findings, we hypothesized that the NF‐κB pathway is involved in *LCN2*‐dependent *CCL5* regulation in GC cells. To test this hypothesis, we first performed Gene Set Enrichment Analysis (GSEA) of TCGA‐STAD samples, which revealed a negative correlation between *LCN2* expression and NF‐κB pathway activation, suggesting that *LCN2* inhibits the NF‐κB pathway (Figure S4A, Supporting Information). Luciferase reporter assays further demonstrated that *LCN2* silencing enhanced NF‐κB‐induced luciferase activity in GC cells, whereas *LCN2* overexpression attenuated it (**Figure** **4**A). Immunofluorescence staining and subcellular fractionation assays showed that *LCN2* overexpression inhibited the nuclear translocation of NF‐κB/p65, whereas *LCN2* silencing promoted it (Figure 4B; Figure S4B, Supporting Information). WB analysis further revealed that *LCN2* overexpression reduced the phosphorylation of IKKα/β, IκBα, and p65, without significantly affecting their total protein levels (Figure 4C). Conversely, *LCN2* silencing increased the phosphorylation of these components (Figure 4C). To further confirm that NF‐κB pathway activation is necessary for *LCN2*‐dependent *CCL5* expression, we treated GC cells with Bay11‐7082, an NF‐κB pathway inhibitor. This treatment blocked the effects of *LCN2* silencing on NF‐κB pathway activation and *CCL5* expression (Figure 4D,E). In summary, our results demonstrate that *LCN2* overexpression inhibits NF‐κB pathway activation and that this pathway mediates the regulatory effect of *LCN2* on *CCL5* expression in GC.

Next, to elucidate the specific mechanism by which *LCN2* inhibits NF‐κB pathway activation, we performed mass spectrometry to identify proteins that interact with *LCN2*. *ANXA1*, a protein known to regulate the NF‐κB pathway by modulating the polyubiquitination of NEMO,^[^ ^36^, ^37^ ^]^ was identified as an *LCN2*‐interacting protein (Figure S4C,D and Table S11, Supporting Information). Subsequently, we confirmed the interactions among *LCN2*, *ANXA1*, and NEMO using exogenous co‐immunoprecipitation (Co‐IP) assays (Figure 4F; Figure S4E, Supporting Information). Furthermore, *ANXA1* knockdown attenuated the *LCN2*‐mediated inhibition of NF‐κB/p65 luciferase activity (Figure 4G); however, the protein levels of *ANXA1* and NEMO remained unchanged following *LCN2* overexpression or silencing (Figure S4F, Supporting Information). Previous studies have demonstrated that NEMO polyubiquitination, particularly Lys63‐linked (K63‐Ub) and Met1‐linear (M1‐Ub) ubiquitination, plays a critical role in NF‐κB pathway activation.^[^ ^36^, ^37^ ^]^ We observed that *LCN2* overexpression reduced K63‐Ub and M1‐Ub levels, whereas *LCN2* silencing increased these levels (Figure 4H). Notably, *ANXA1* knockdown rescued the reduction in K63‐Ub and M1‐Ub levels observed with *LCN2* overexpression (Figure 4I). Similarly, Co‐IP assays further confirmed that *LCN2* silencing increased the K63‐Ub and M1‐Ub levels of NEMO, whereas *LCN2* overexpression reduced them (Figure 4J). Moreover, *ANXA1* knockdown attenuated the *LCN2*‐induced decrease in NEMO polyubiquitination (Figure 4K). These findings suggest that *LCN2* regulates NEMO polyubiquitination through *ANXA1*, thereby inhibiting the activation of the NF‐κB pathway. The linear ubiquitin assembly complex (LUBAC), composed of heme‐oxidized IRP2 ubiquitin ligase‐1L (HOIL‐1), ring finger protein 31 (HOIP), and shank‐associated RH domain interactor (Sharpin), plays a crucial role in K63‐ and M1‐linked polyubiquitination of NEMO.^[^ ^36^, ^37^ ^]^ Meanwhile, we found that the expression levels of HOIP, HOIL‐1, and Sharpin decreased with *LCN2* overexpression but increased with *LCN2* silencing (Figure 4L). Furthermore, *ANXA1* knockdown mitigated the *LCN2*‐induced downregulation of HOIP, HOIL‐1, and Sharpin (Figure 4M). Collectively, these results demonstrate that *LCN2* attenuates NF‐κB pathway activation by inhibiting K63‐ and M1‐linked ubiquitination of NEMO in an *ANXA1*‐dependent manner, thereby downregulating *CCL5* expression in GC cells.

### 2.5 CCL5‐Activated TAMs Induce Lymphangiogenesis and Lymph‐Node Metastasis Through the VEGFC/VEGFR3 Pathway

**Figure 5:**
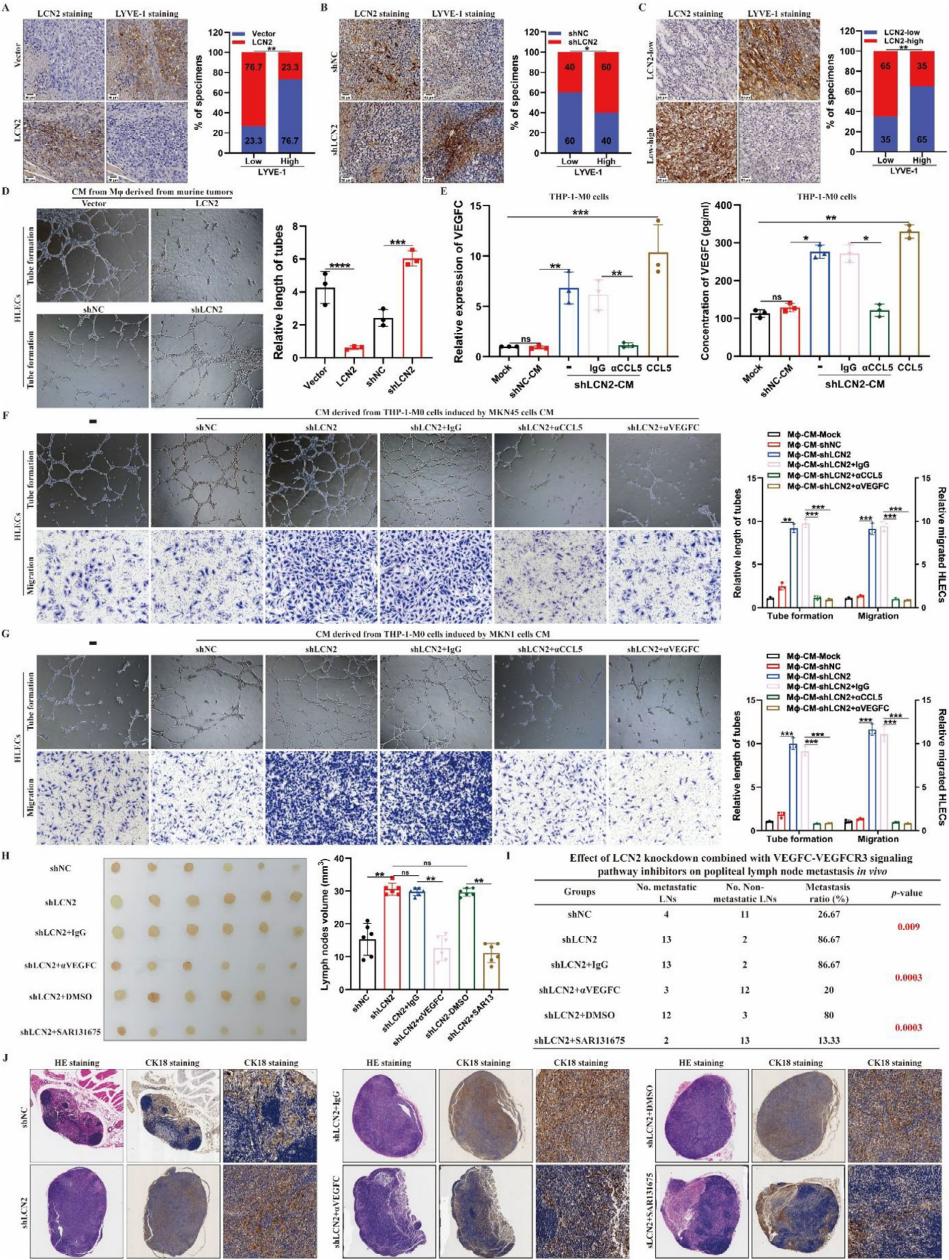
CCL5‐activated TAMs induce lymphangiogenesis and lymph‐node metastasis through the VEGFC/VEGFR3 pathway. **A,B)** Representative images (left) and percentages (right) of LCN2 expression and LYVE‐1‐indicated lymphatic vessels in primary tumor tissue of mouse footpads from the indicated groups. **C)** Representative images (left) and percentages (right) of LCN2 expression and LYVE‐1‐indicated lymphatic vessels in human primary gastric cancer tissues. **D)** Representative images (left) and quantifications (right) of tube formation by MLECs treated with conditioned medium collected from TAMs sorted from the indicated murine tumors. **E)** RT‐qPCR (left) and ELISA (right) analyses of VEGFC expression in THP‐1‐derived M0 cells treated with conditioned medium collected from the indicated GC cells. **F,G)** Representative images (left) and quantifications of tube formation and transwell assays by HLECs treated with conditioned medium collected from THP‐1‐derived M0 macrophages induced by the indicated gastric cells with the corresponding treatment. **H)** Representative images (left) and volumes (right) of the enucleated popliteal LNs in the indicated mouse groups. **I)** Ratios of metastatic and non‐metastatic enucleated popliteal LNs to the total count for the indicated groups. **J)** Representative images of HE and CK‐18 IHC staining of the popliteal lymph nodes for the indicated groups. Data are expressed as the mean ± SD of biological replicate experiments. * indicates *p *< 0.05, ** indicates *p *< 0.01, *** indicates *p *< 0.001, **** indicates *p *< 0.0001, and ns indicates no significance.

Tumor‐associated lymphangiogenesis is considered a rate‐limiting step in LN metastasis.^[^ ^38^ ^]^ As *LCN2* expression was significantly correlated with LN metastasis in GC, we examined the effect of LCN2 on lymphangiogenesis in GC tissue. IHC analyses revealed a negative correlation between *LCN2* expression and lymphatic vessel density in both mouse and human GC tissue, as indicated by the expression level of lymphatic vessel endothelial hyaluronan receptor 1 (*LYVE‐1*). These findings suggest that *LCN2* may inhibit lymphangiogenesis in vivo (**Figure** **5**A–C). Further analysis of TCGA‐STAD samples revealed that *LCN2* expression was negatively correlated with *LYVE‐1* and *VEGFC* expression, whereas *CCL5* expression was positively correlated with *LYVE‐1* and *VEGFC* expression (Figure S5A, Supporting Information). However, in vitro human lymphatic endothelial cell (HLEC) tube formation and transwell assays showed that CM from GC cells with *LCN2* overexpression or silencing alone had minimal effects on lymphangiogenesis (Figure S5B,C, Supporting Information), indicating that additional factors may contribute to *LCN2*‐dependent lymphangiogenesis in vivo. Furthermore, we found that neither overexpression nor silencing of LCN2 in GC cells or treatment with CCL5 affected their own VEGFC expression or secretion (Figure S5D–I, Supporting Information). This further suggests that other factors within the TME regulate VEGFC expression, thereby promoting lymphangiogenesis and LN metastasis. Various tumor‐associated immune cells, including TAMs, cancer‐associated fibroblasts, mast cells, and neutrophils, play crucial roles in lymphangiogenesis.^[^ ^16^, ^17^, ^18^, ^23^ ^]^ Furthermore, given that our results indicate *LCN2* inhibits LN metastasis of GC via *CCL5*‐dependent macrophage activation, we hypothesized that TAMs may also be involved in *LCN2*‐dependent lymphangiogenesis. Supporting this, we found that CM from TAMs isolated from *LCN2*‐silenced murine tumors significantly enhanced lymphatic vessel formation, whereas CM from TAMs isolated from *LCN2*‐overexpressing murine tumors inhibited it (Figure 5D). These findings suggest that *LCN2* silencing in GC cells promotes TAM‐dependent lymphangiogenesis. VEGFC, a key lymphangiogenic factor, is critical for increasing lymphatic vessel abundance around tumors and promoting LN metastasis in solid tumors.^[^ ^15^ ^]^ Notably, database analysis revealed a significant negative correlation between *LCN2* and *VEGFC* expression (Figure S5A, Supporting Information). To determine whether VEGFC is involved in *LCN2*‐dependent lymphangiogenesis and whether GC cell‐secreted *CCL5* induces *VEGFC* upregulation in TAMs, we performed RT‐qPCR and ELISA. The results showed that *VEGFC* expression and secretion in TAMs were upregulated following treatment with CM from *LCN2*‐silenced GC cells or recombinant *CCL5*, and this effect was prevented by *CCL5*‐neutralizing antibodies (Figure 5E). These findings suggest that TAMs activated by *LCN2*‐silencing‐induced *CCL5*, contribute to pro‐lymphangiogenic processes by producing *VEGFC*. Additionally, CM from TAMs co‐cultured with CM from *LCN2*‐silenced GC cells significantly enhanced HLEC tube formation and motility, whereas treatment with neutralizing antibodies against *CCL5* or *VEGFC* reversed these effects (Figure 5F,G). This indicates that blocking the *CCL5/TAM/VEGFC* axis could inhibit *LCN2*‐dependent lymphangiogenesis in GC. Furthermore, in vivo assays demonstrated that blocking *VEGFC/VEGFR3* signaling using a *VEGFC*‐neutralizing antibody or a specific VEGFR3 inhibitor (SAR131675) rescued the enhancement of LN metastasis induced by *LCN2* silencing, as evidenced by reduced LN volume and a decreased lymphatic metastasis ratio (Figure 5H–J). Collectively, these results suggest that TAMs activated by *LCN2*‐regulated *CCL5* promote lymphangiogenesis and LN metastasis through *VEGFC/VEGFR3* signaling in GC.

### 2.6 CCL5‐CCR5 Axis Activates the PI3K/AKT/GSK3β Pathway to Repolarize TAMs and Enhance Their VEGFC/IL‐10 Expression

**Figure 6:**
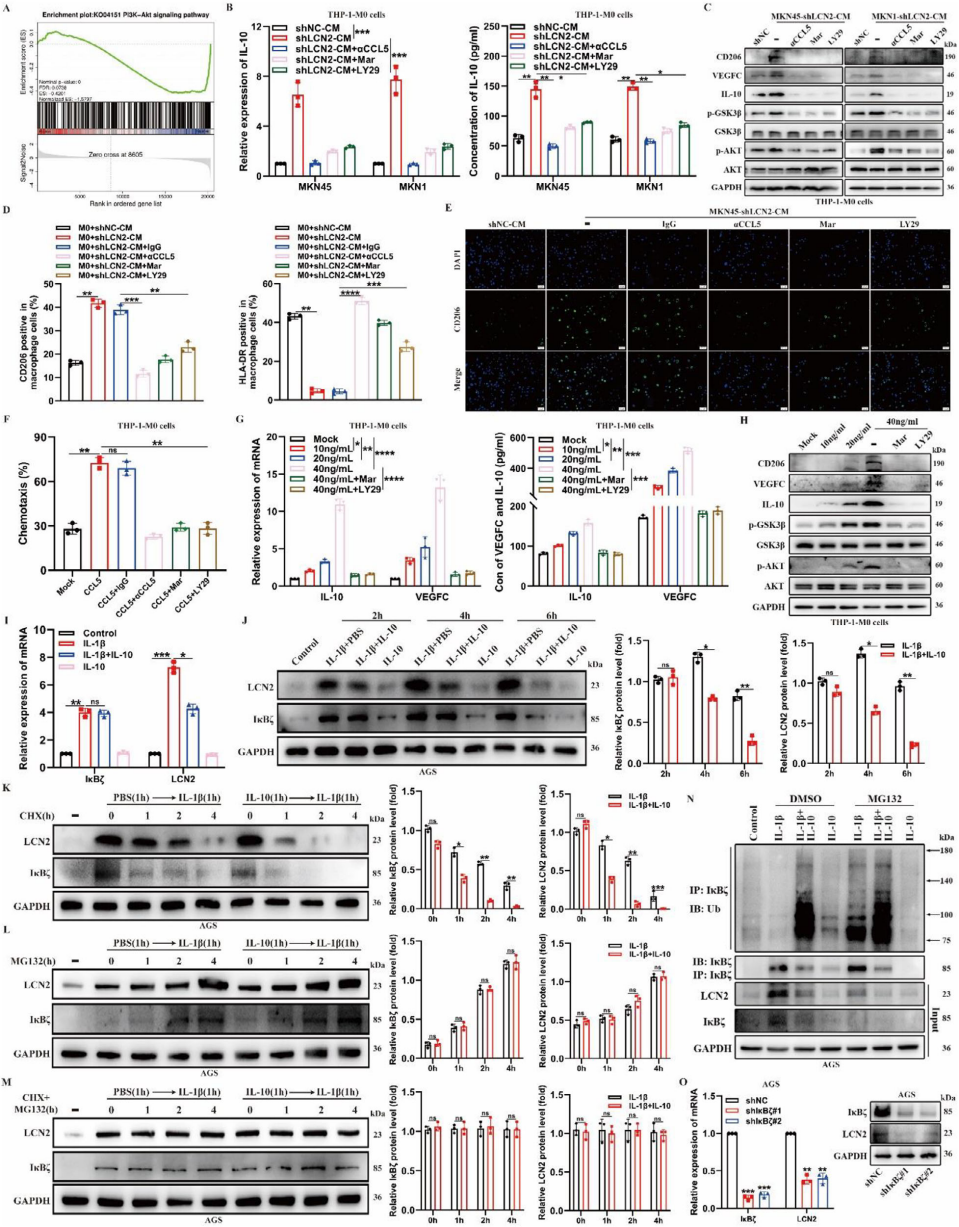
A positive‐feedback loop of the CCL5/CCR5/PI3K/AKT/GSK3β/IL‐10/IκBζ/LCN2 axis is formed between gastric cancer cells and tumor associated‐macrophages. **A)** GSEA reveals the PI3K/AKT pathway associated with CCL5‐induced macrophage activation. **B)** RT‐qPCR (left) and ELISA (right) analyses of IL‐10 expression in THP‐1‐derived M0 macrophages treated with the conditioned medium collected from the indicated GC cells combined with CCL5‐neutralizing antibody, Mar, or LY29. **C)** Western blot analysis of the expression of the corresponding proteins in THP‐1‐derived M0 macrophages treated with the conditioned medium collected from the indicated GC cells combined with CCL5‐neutralizing antibody, Mar, or LY29. **D)** Quantitative results of CD206^+^ cells and HLA‐DR^+^ cells in THP‐1‐derived M0 macrophages with the corresponding treatment. **E)** Representative immunofluorescence staining images for CD206 in THP‐1‐derived M0 macrophages with the corresponding treatment. **F)** Chemotactic abilities of THP‐1‐derived M0 macrophages with the corresponding treatment assessed using transwell assays. **G)** RT‐qPCR (left) and ELISA (right) analyses of IL‐10 and VEGFC expression in THP‐1‐derived M0 macrophages treated with different concentrations of CCL5 combined with Mar or LY29. **H)** Western blot analysis of the expression of the corresponding proteins in THP‐1‐derived M0 macrophages treated with different concentrations of CCL5 combined with Mar or LY29. **I)** RT‐qPCR analysis of NFKBIZ and LCN2 mRNA expression at 6 h. **J)** Western blot analysis (left) and relative quantification (right) of IκBζ and LCN2 protein expression in serum‐starved AGS cells with the corresponding treatment. **K–M)** Western blot analysis (left) and relative quantification (right) of IκBζ and LCN2 protein expression in serum‐starved AGS cells treated with PBS, IL‐10, IL‐1β, or various inhibitors as indicated. **N)** Western blot analysis of IκBζ ubiquitin levels in serum‐starved AGS cell lysates with the corresponding treatment, following pretreatment with PBS or IL‐10 for 1.5 h and subsequent stimulation with PBS or IL‐1β in the presence or absence of MG132 for 3 h. **O)** RT‐qPCR and western blot assay analyses of LCN2 expression in IκBζ‐silenced and control GC cells, as indicated. Data are expressed as mean ± SD of biological replicate experiments. * indicates *p* < 0.05, ** indicates *p *< 0.01, *** indicates *p *< 0.001, **** indicates *p *< 0.0001, and ns indicates no significance.

To further investigate the mechanisms underlying *CCL5*‐mediated TAM recruitment and M2‐type polarization, we performed transcriptome sequencing of THP‐1‐derived M0 macrophages treated with PBS or *CCL5*. The results showed significant activation of the PI3K/AKT/GSK3β pathway, which is closely associated with macrophage activation,^[^ ^39^ ^]^ following *CCL5* treatment (**Figure** **6**A; Figure S6A and Table S12, Supporting Information). Furthermore, it is well established that the *CCL5*‐*CCR5* axis can activate the PI3K/AKT/GSK3β pathway.^[^ ^40^ ^]^ Based on this, we speculated that this activation might be involved in *CCL5*‐mediated macrophage recruitment and M2‐type polarization. RT‐qPCR, ELISA, and WB analyses showed that *CD206*, *VEGFC*, and *IL‐10* expression, as well as the activation of AKT and GSK3β, were significantly increased in THP‐1‐derived M0 macrophages after treatment with CM from *LCN2*‐silenced GC cells (Figure 6B,C). Moreover, these effects were reversed by treatment with *CCL5*‐neutralizing antibodies, the CCR5 inhibitor Maraviroc (Mar), or the PI3K inhibitor LY294002 (LY29) (Figure 6B,C). Flow cytometry and immunofluorescence analyses further confirmed these findings (Figure 6D,E; Figure S6C, Supporting Information). Meanwhile, both *CCL5*‐neutralizing antibodies and CCR5 and PI3K inhibitors significantly reduced the *CCL5*‐induced enhancement of THP‐1‐derived M0 macrophage recruitment (Figure 6F; Figure S6B, Supporting Information). Similarly, *CCL5* increased *IL‐10* and *VEGFC* expression and secretion while activating the PI3K/AKT/GSK3β pathway in TAMs in a concentration‐dependent manner. However, these effects were mitigated by either CCR5 or PI3K inhibition (Figure 6G,H). More importantly, in vivo assays demonstrated that blocking the PI3K/AKT/GSK3β pathway using a specific PI3K and AKT inhibitor (LY294002; MK‐2206) rescued the enhancement of LN metastasis induced by *LCN2* silencing, as evidenced by reduced LN volume and a decreased lymphatic metastasis ratio (Figure S6D–F, Supporting Information). Taken together, these results suggest that the *CCL5‐CCR5* axis induces the recruitment and M2‐type polarization of TAMs and promotes their *VEGFC* and *IL‐10* expression through activation of the PI3K/AKT/GSK3β pathway.

### 2.7 TAMs‐Derived IL‐10 Inhibits the Expression of IκBζ and Its Target Gene LCN2 in GC Cells by Promoting IκBζ Degradation

IL‐20 has been reported to downregulate *LCN2* expression in hepatocytes by promoting IκBζ degradation.^[^ ^41^ ^]^ IL‐10 and IL‐20 belong to the IL‐10 family and share similar biological functions.^[^ ^42^ ^]^ Furthermore, analysis of TCGA‐STAD samples revealed a significant negative correlation between *IL‐10* and *LCN2* expression, as well as a positive correlation between *IL‐10* and *CCL5* expression (Figure S7A, Supporting Information). These findings led us to hypothesize that TAM‐derived *IL‐10* may inhibit *LCN2* expression by modulating IκBζ, thereby establishing a positive‐feedback loop. IκBζ, an inducible nuclear protein encoded by *NFKBIZ*, is rapidly upregulated in response to pro‐inflammatory stimuli such as *IL‐1β* and plays a critical role in regulating the expression of several downstream target genes, including *LCN2*, *CCL20*, *CSF3*, and S100 family proteins.^[^ ^41^ ^]^ To explore whether *IL‐10* regulates *LCN2* expression, we conducted in vitro experiments using AGS cells, following a previously described method (Figure S7B, Supporting Information). The results showed that *IL‐1β* treatment significantly upregulated *IκBζ* and *LCN2* mRNA and protein levels (Figure 6I,J). Notably, pre‐treatment with *IL‐10* reduced *IL‐1β*‐induced *IκBζ* protein levels without affecting *NFKBIZ* mRNA levels, whereas both *LCN2* protein and mRNA levels were significantly decreased (Figure 6I,J). This suggests that *IL‐10* regulates *IκBζ* protein stability. To investigate whether IL‐10 could regulate IκBζ protein stability in GC cells, we assessed *IκBζ* protein degradation over time using cycloheximide (CHX), a de novo protein synthesis inhibitor. As expected, CHX treatment promoted *IL‐1β*‐induced *IκBζ* degradation in a time‐dependent manner (Figure 6K). However, the rate of degradation was significantly accelerated with *IL‐10* pre‐treatment (Figure 6K), suggesting that *IL‐10* pre‐treatment enhances *IκBζ* degradation. Given that *IκBζ* degradation is known to occur via ubiquitin‐mediated proteasomal pathways, we next examined the role of ubiquitination in this process. Treatment with the proteasome inhibitor MG132 prevented *IκBζ* degradation, regardless of *IL‐10* pre‐treatment (Figure 6L,M). Furthermore, *IL‐10* treatment enhanced *IL‐1β*‐induced *IκBζ* ubiquitination even in the presence of MG132, suggesting that *IL‐10* promotes *IκBζ* degradation via increased ubiquitination (Figure 6N). Meanwhile, we also confirmed that *IκBζ* knockdown inhibited *LCN2* expression in GC cells (Figure 6O). Finally, in vivo assays demonstrated that IL‐10 inhibition with the *IL‐10*‐neutralizing antibody partially blocked the increase in lymphatic metastasis induced by LCN2 silencing (Figure S7C–E, Supporting Information). Collectively, these findings indicate that TAM‐derived *IL‐10* inhibits the expression of IκBζ and its downstream gene *LCN2* in GC cells by promoting *IκBζ* degradation, thereby establishing a positive feedback loop between GC cells and TAMs.

### 2.8 Clinical Relevance of the LCN2‐CCL5‐TAM‐VEGFC‐Lymphangiogenesis Axis in GC

**Figure 7:**
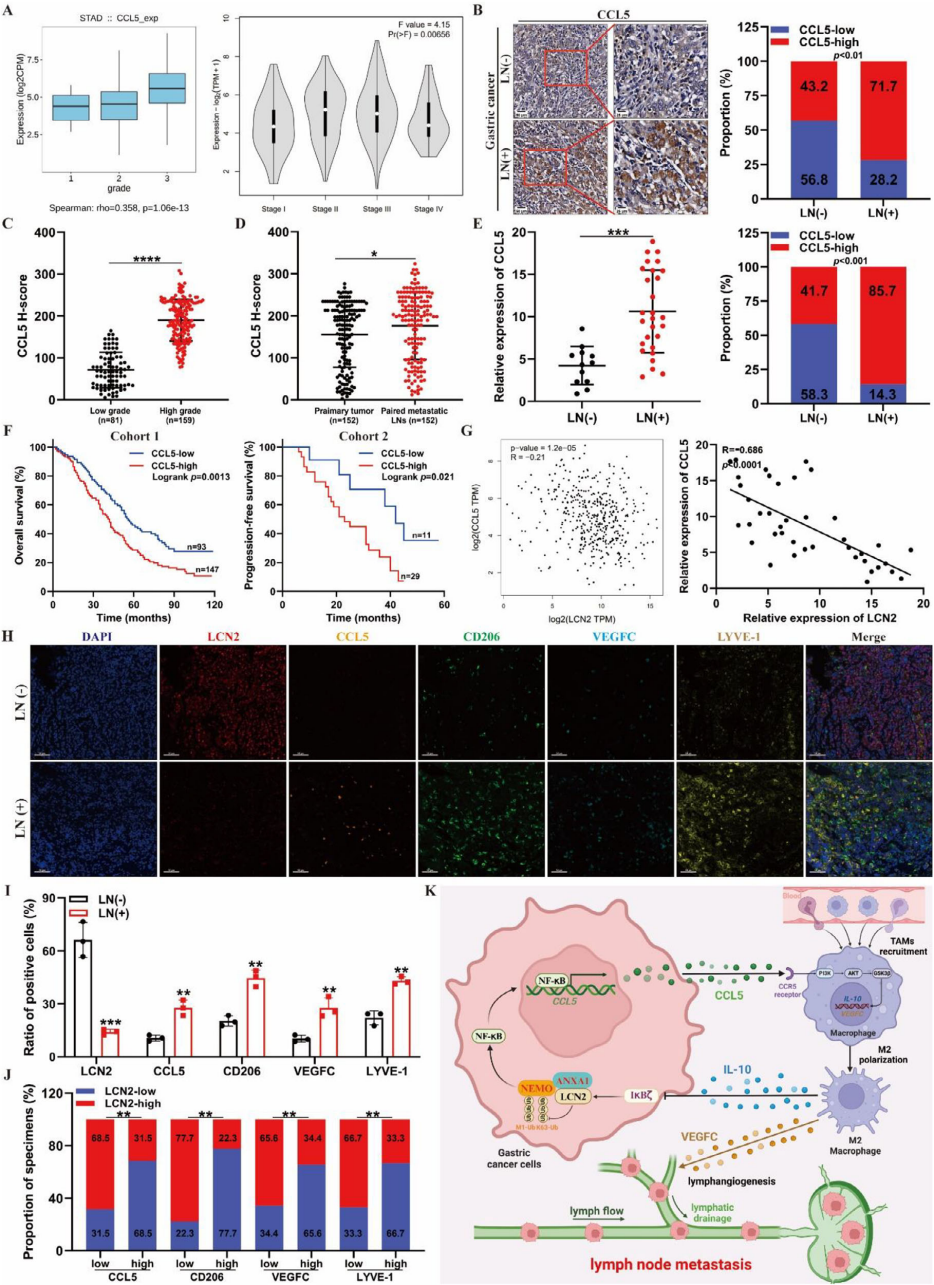
Clinical relevance of the LCN2‐CCL5‐TAM‐VEGFC‐lymphangiogenesis axis in GC. **A)** Correlation between CCL5 expression in gastric cancer tissues from TCGA‐STAD and the pathological grades and clinical stages of patients with gastric cancer. **B)** Representative IHC images (left) and percentages (right) of CCL5 expression in paraffin‐embedded sections of gastric cancer tissues with (*n* = 152) or without (*n* = 88) LN metastasis. **C)** Correlation of CCL5 expression in primary gastric cancer tissues (*n* = 240) assessed by IHC with pathological grade. **D)** IHC analyses of CCL5 expression in primary gastric cancer tissues versus paired metastatic LN tissues (*n* = 152). **E)** RT‐qPCR analyses (left) and percentages (right) of CCL5 expression in freshly collected gastric cancer tissues with (*n* = 28) or without (*n* = 12) LN metastasis. **F)** Kaplan‐Meier survival analyses of OS (left) and PFS (right) for patients with GC with low versus high CCL5 expression. **G)** Correlation analyses of LCN2 and CCL5 mRNA expression in the TCGA database and our clinical cohort (*n* = 40). **H)** Representative immunofluorescence images (left) for LCN2, CCL5, CD206, VEGFC, and LYVE‐1 in paraffin‐embedded sections of gastric cancer tissues with and without LN metastasis. **I)** Ratios of LCN2^+^ cells, CCL5^+^ cells, CD206^+^ cells, VEGFC^+^ cells, and LYVE‐1^+^ cells in paraffin‐embedded sections of gastric cancer tissues with and without LN metastasis. **J)** Quantification of high or low expression levels of CCL5, CD206, VEGFC, and LYVE‐1 in gastric cancer tissues with different LCN2 expression levels. **K)** Illustrative model showing the proposed mechanism by which an LCN2‐dependent positive‐feedback loop between gastric cancer cells and tumor‐associated macrophages mediates lymphangiogenesis and lymphatic metastasis. Data are expressed as mean ± SD of biological replicate experiments. * indicates *p* < 0.05, ** indicates *p *< 0.01, *** indicates *p *< 0.001, and **** indicates *p *< 0.0001.

Finally, we assessed the clinical significance of the *LCN2*‐*CCL5*‐TAM‐*VEGFC*‐lymphangiogenesis axis in GC. First, we examined the relationship between *CCL5* expression and pathological grades as well as clinical stages in TCGA‐STAD samples. The analysis revealed that higher *CCL5* expression was associated with higher tumor grades and advanced stages of GC (**Figure** **7**A). Next, we measured *CCL5* protein levels in our clinical GC cohorts using IHC. The results showed a significant positive correlation between *CCL5* protein levels and LN metastasis (Figure 7B,D; Table S2, Supporting Information). Similarly, elevated *CCL5* expression correlated with higher tumor grades (Figure 7C; Table S2, Supporting Information), supporting its potential role as a biomarker for disease progression. Moreover, RT‐qPCR analysis demonstrated that *CCL5* mRNA levels were significantly upregulated in fresh GC tissue with LN metastasis (Figure 7E). Kaplan‐Meier survival analysis further revealed that higher *CCL5* expression was associated with poorer OS and PFS in patients with GC (Figure 7F), suggesting that *CCL5* not only contributes to GC progression but also serves as a prognostic marker. More importantly, analysis of *LCN2* and *CCL5* mRNA expression levels in GC tissue from both TCGA databases and our clinical cohorts revealed a negative correlation between *LCN2* and *CCL5* expression (Figure 7G). Multicolor immunofluorescence assays further revealed that GC tissue with LN metastasis showed higher *CCL5, CD206, VEGFC*, and *LYVE‐1* expression and lower *LCN2* expression than GC tissue without LN metastasis (Figure 7H,I). Additionally, lower *LCN2* levels were associated with increased expression of *CCL5*, *CD206*, *VEGFC*, and *LYVE‐1* (Figure 7J). Taken together, these findings demonstrate that *LCN2*‐regulated *CCL5* reshapes the GC microenvironment by regulating TAM infiltration and *VEGFC* secretion, ultimately driving lymphangiogenesis and lymphatic metastasis.

## 3 Discussion

LN metastasis is a key predictor of poor prognosis in patients with GC and presents significant challenges for clinical treatment.^[^ ^43^ ^]^ Therefore, exploring the molecular mechanisms underlying LN metastasis and identifying novel therapeutic targets is crucial for improving patient outcomes. Lymphangiogenesis, the formation of new lymphatic vessels, plays a pivotal role in LN metastasis by facilitating the dissemination of cancer cells through the lymphatic system.^[^ ^8^, ^9^, ^10^, ^11^ ^]^ However, the precise mechanisms driving lymphangiogenesis in GC remain largely unknown. In this study, we identified *LCN2* as a critical regulator that inhibits lymphangiogenesis and LN metastasis in GC by modulating the TME. *LCN2*, known as neutrophil gelatinase‐associated lipocalin, is expressed in various tissues and cell types.^[^ ^44^ ^]^ It functions as both an intracellular and secreted protein and has been widely studied for its diverse and complex functions in multiple diseases, including cancers.^[^ ^24^ ^]^ However, different studies on *LCN2* have reported conflicting conclusions regarding its role in tumor progression and metastasis. Additionally, the effects of *LCN2* on lymphangiogenesis and TME modulation have not been previously reported. Here, we revealed that *LCN2* expression is significantly downregulated in metastatic GC cells. Moreover, low expression of LCN2 was associated with increased lymphangiogenesis, higher LN metastasis rates, and shorter patient survival. Collectively, these findings suggest that *LCN2* may serve as a novel and promising diagnostic and therapeutic target for clinically identifying and controlling LN metastasis in GC.

TAMs, a predominant component of the TME, play multifaceted roles in tumor progression and metastasis.^[^ ^20^, ^21^ ^]^ Specifically, TAMs exhibit remarkable plasticity and are highly responsive to variations in the TME, typically classified into M1 and M2 subtypes. Among these, M2‐type TAMs generally promote tumor growth and metastasis, whereas M1‐type TAMs exert inhibitory effects.^[^ ^45^ ^]^ Notably, M2‐type TAMs have been implicated in lymphangiogenesis and LN metastasis.^[^ ^22^, ^23^ ^]^ However, the precise role of M2‐type TAMs in lymphangiogenesis and LN metastasis in GC, as well as the underlying molecular mechanisms, remains largely unclear. In this study, we demonstrated that M2‐type TAMs are integral to *LCN2*‐mediated inhibition of lymphangiogenesis and LN metastasis in GC. We further found that *LCN2* silencing promotes the recruitment and M2‐type polarization of TAMs by upregulating *CCL5* expression and secretion. Additionally, *CCL5*‐mediated reprogramming of TAMs enhances lymphangiogenesis and LN metastasis by increasing *VEGFC* production. Overall, our findings provide novel insights into the specific mechanisms by which M2‐type TAMs contribute to lymphangiogenesis and LN metastasis in GC.

VEGFC, a key regulator of lymphatic network expansion in the TME, promotes VEGFR3‐dependent lymphatic vessel growth during LN metastasis of tumors.^[^ ^15^, ^46^ ^]^ Recent studies have shown that targeting the VEGFC/VEGFR3 pathway with inhibitors can obstruct lymphatic vessel development, growth, and regeneration, making it a promising therapeutic strategy for limiting LN metastasis in tumors.^[^ ^47^ ^]^ Notably, several small‐molecule inhibitors targeting VEGFC and VEGFR3 have already entered clinical trials.^[^ ^48^, ^49^ ^]^ In this study, we found that VEGFC expression in TAMs was significantly upregulated in response to CM from *LCN2*‐silenced GC cells or recombinant CCL5. Mechanistically, the CCL5‐CCR5 axis promotes VEGFC expression and secretion in TAMs by activating the PI3K/AKT/GSK3β pathway. More importantly, blocking VEGFC/VEGFR3 signaling with a VEGFC‐neutralizing antibody or a specific VEGFR3 inhibitor reversed the enhancement of lymphangiogenesis and LN metastasis induced by *LCN2* silencing in GC. Therefore, our findings provide compelling evidence that clinically targeting VEGFC/VEGFR3 signaling could serve as an effective strategy to inhibit LN metastasis in patients with low *LCN2* expression GC.

Another key finding of this study is that *LCN2* silencing promotes *CCL5* expression and secretion in GC cells through transcriptional regulation, leading to the recruitment and M2‐type polarization of TAMs. Chemokines serve as crucial messengers that mediate interactions between tumor cells and TAMs. Our study identified *CCL5* as a key target of *LCN2*‐mediated inhibition of TAM activation and LN metastasis in GC. *CCL5*, secreted by various cell types, is highly expressed in multiple malignancies and plays a critical role in tumor metastasis and progression.^[^ ^50^ ^]^ Additionally, emerging evidence suggests that the *CCL5/CCR5* axis plays an important role in TAM activation;^[^ ^51^, ^52^ ^]^ however, its specific effects on TAMs in GC remain largely unknown. More importantly, some known inhibitors, such as maraviroc, cenicriviroc, and MET‐CCL5, which could prevent the binding of *CCL5* and CCR5, have demonstrated anti‐inflammatory and anti‐cancer effects in several phase I and phase II clinical trials (NCT01736813; NCT00569985; NCT03274804).^[^ ^53^ ^]^ Therefore, elucidating the molecular mechanisms that sustain *CCL5* expression and determining the precise role of the *CCL5/CCR5* axis in TAM activation in GC could contribute to the development of promising therapeutic strategies. Here, we revealed that *LCN2* silencing upregulated *CCL5* expression in GC cells by activating the NF‐κB pathway through ANXA1‐dependent polyubiquitination of NEMO. This upregulation of *CCL5* directly induces the recruitment and M2‐type polarization of TAMs. Furthermore, blocking the *CCL5/CCR5* axis reduced M2‐type TAM infiltration and LN metastasis in *LCN2*‐silenced GC cells, suggesting that inhibiting the *CCL5/CCR5* axis suppresses LN metastasis in *LCN2*‐low‐expressing GC. Therefore, our study reveals the precise mechanism by which *LCN2* silencing activates *CCL5* expression in GC and highlights the therapeutic potential of targeting the *CCL5/CCR5* axis in GC treatment.

The interplay between tumor cells and immune‐infiltrating cells within the TME is typically bidirectional, forming a dynamic feedback loop that regulates tumor progression and metastasis. Tumor cell‐derived factors drive the chemotaxis and differentiation of TAMs, which, in turn, regulate the biological behavior of tumor cells by secreting various cytokines. In our study, we observed that *IL‐10* expression and secretion were significantly increased in TAMs treated with CM from *LCN2*‐silenced GC cells or recombinant *CCL5*. *IL‐10*, a cytokine secreted by various immune cells, including macrophages, plays a central role in immune regulation.^[^ ^42^ ^]^ Furthermore, TAM‐derived *IL‐10* has been shown to promote tumor progression through multiple mechanisms.^[^ ^54^ ^]^ Interestingly, *IL‐20*, another member of the *IL‐10* superfamily, has been previously reported to inhibit *LCN2* expression in hepatocytes by promoting *IκBζ* degradation.^[^ ^41^ ^]^ Here, we demonstrated that *IL‐10* similarly suppresses *LCN2* expression in GC cells by promoting *IκBζ* degradation. This *IL‐10*‐mediated suppression of *LCN2* expression creates a positive feedback loop between GC cells and TAMs, thereby continuously promoting lymphangiogenesis and LN metastasis. Therefore, disrupting this loop may represent a promising therapeutic strategy for inhibiting LN metastasis in GC.

In conclusion, our study highlights the crucial role of *LCN2* in inhibiting lymphangiogenesis and LN metastasis in GC through a complex feedback mechanism involving *TAM* polarization and *CCL5* regulation. These insights into the molecular mechanisms driving lymphatic metastasis in GC provide valuable information for developing targeted therapies aimed at positive feedback loop modulation. Understanding how *LCN2* influences *CCL5* expression and *TAM* activation could guide the development of promising therapeutic strategies to inhibit LN metastasis and improve the prognosis of patients with GC.

## 4 Experimental Section

### Clinical Samples

In this study, a total of 240 GC tissue samples and 152 corresponding LN tissue samples were collected from the First Affiliated Hospital of Sun Yat‐sen University for immunohistochemical examination from 2014 to 2020. Moreover, 40 fresh GC tissues were obtained for RT‐qPCR from 2020 to 2022. All specimens were collected with the written informed consent form the patients. The experiments strictly adhered to the principles of the Declaration of Helsinki and were approved by the Ethics Committees of the First Affiliated Hospital of Sun Yat‐sen University (project number: 2020–164).

### Cell Lines and Cell Culture

Human GC cell lines AGS (RRID: CVCL_0139), MKN1 (RRID: CVCL_1415), and MKN45 (RRID:CVCL_0434), along with the mouse forestomach carcinoma cell line (MFC, RRID: CVCL_5J48) were obtained from the Institute of Cell Biology, Chinese Academy of Sciences (Shanghai, China). The RAW264.7 (RRID:CVCL_0493) mouse macrophage cell line was purchased from the American Type Culture Collection (ATCC, Manassas, VA, USA). AGS cells were cultured in Dulbecco's modified Eagle's medium (Gibco, Waltham, MA, USA), while MKN1, MKN45, MFC, and RAW264.7 cells were maintained in Roswell Park Memorial Institute‐1640 medium (Gibco). All media were supplemented with 10% fetal bovine serum (FBS; Gibco) and 1% penicillin/streptomycin (Gibco). Human lymphatic endothelial cells (HLECs, Catalog #2500) were obtained from ScienCell Research Laboratories (San Diego, CA, USA) and cultured in Endothelial Cell medium (Gibco) containing 5% FBS. All cells were incubated in a humidified atmosphere of 5% CO_2_ at 37 °C and were confirmed negative for mycoplasma contamination using PCR with Mycoplasma PCR Detection Kit (Beyotime).

### Popliteal Lymphatic Metastasis Model

A popliteal lymphatic metastasis model was used to simulate LN metastasis in GC, constructed as described previously.^[^ ^16^, ^23^ ^]^ Briefly, 5‐week‐old 615‐line mice were obtained from the Experimental Animal Center of Sun Yat‐sen University (Guangzhou, Guangdong, China) and raised in pathogen‐free conditions. A total of 1 × 10^6^ MFC cells were collected, resuspended in 30 µL of phosphate‐buffered saline (PBS), and slowly injected into the footpads of the mice. When the footpad tumor reached a volume of 800 mm^3^, the tumors and popliteal LNs were excised for further analysis. The Animal Care and Use Committee of Sun Yat‐sen University approved all animal care and experimental procedures (approval number: [2025]030). Furthermore, our study examined male and female animals, and similar findings are reported for both sexes.

### Construction of Stable Cell Lines and Cell Transfection

The lentivirus, including short hairpin RNA (shRNA) silencing LCN2, CCL5, and ANXA1, as well as overexpressing LCN2, was synthesized by iGene Biotechnology (Guangzhou, Guangdong, China). A scramble shRNA vector and an empty vector were used as a negative control. The corresponding lentiviruses were used to infect cells according to the manufacturer's protocols. Next, the infected cells were selected using puromycin or hygromycin for 2 weeks. Overexpression plasmids were constructed using pEZ‐Lv201 (iGene Biotechnology), and shRNA constructs were created with LVRU6GP (iGene Biotechnology). Lipofectamine 3000 (Invitrogen, Carlsbad, CA, USA) was utilized for cell transfection following the manufacturer's instructions. Table S3 (Supporting Information) lists the shRNA and siRNA sequences.

### Transcriptome Sequencing (RNA‐Seq)

Total RNA was extracted from tissues and cells using TRIzol reagent (Invitrogen), followed by transcriptome sequencing (RNA‐seq) as outlined in the manufacturer's instructions. Briefly, the extracted RNA was quantified and qualified using an Agilent 2100 Bioanalyzer (Agilent, Santa Clara, CA, USA) and Qubit 3.0 fluorometer (Invitrogen). Stranded sequencing libraries were prepared with the KC‐Digital Stranded mRNA Library Prep Kit (Invitrogen), and RNA‐seq was performed on the Illumina HiSeq 4000 platform at Guangzhou Gidio Company.

### THP‐1 Cell Culture and Macrophage Induction

The human monocyte cell line THP‐1 (RRID:CVCL_0006) was sourced from Procell Life Science & Technology Co., Ltd. (Wuhan, Hubei, China) and cultured in RPMI‐1640 medium supplemented with 10% FBS and 0.05 mm β‐mercaptoethanol (Sigma, St. Louis, MO, USA). THP‐1 cells were differentiated into macrophages (M0, Mφ) by treatment with 100 ng mL^−1^ phorbol‐12‐myristate 13‐acetate (PMA; Sigma) for 24 h. M0 macrophages were further stimulated with IFN‐γ (20 ng mL^−1^) and LPS (100 ng mL^−1^) for 48 h to generate M1‐type macrophages, or with IL‐4 (20 ng mL^−1^) and IL‐13 (20 ng mL^−1^) for 48 h to induce M2‐type macrophages.

### Chemotaxis and Co‐Culture Assays

The assays of M0 macrophage chemotaxis and co‐culture of tumor cells and M0 macrophages were performed using transwell chambers (8 µm inserts; Corning, Tewksbury, MA, USA) in 24‐well plates (Figure S2G, Supporting Information). For the chemotaxis assay, THP‐1‐derived M0 cells were added to the upper chamber, and corresponding GC cells or CM, with or without neutralizing antibodies, were added to the lower compartment. After a 4 h incubation at 37 °C, migrated cells were fixed and stained. Finally, stained cells were captured using a microscope (Olympus, Tokyo, Japan) and quantified, with mean values calculated for analysis. In the co‐culture model, 5 × 10^4^ GC cells were placed in the upper chamber, and an equal number of M0 macrophages (PMA‐treated THP‐1 or RAW264.7 cells) were cultured in the lower chamber for 48 h. Cells from the lower chamber were then collected for protein and RNA extraction, with M1 and M2 marker expression analyzed via RT‐qPCR and flow cytometry.

### Flow Cytometry Analysis

Flow cytometry was applied to analyze the expression of cell‐surface (CD45, CD3, CD4, CD8, F4/80, CD68, HLA‐DR) and intracellular (CD206) markers. For THP‐1‐derived M0 and RAW264.7 cells, they were collected, washed, and resuspended in staining buffer (2% FBS in PBS). Next, these cells were blocked with 3% bovine serum albumin (Beyotime, Guangzhou, Guangdong, China) and stained with PE anti‐human/mouse CD206 and FITC anti‐human/mouse HLA‐DR. Tumor and spleen tissue were digested by collagenase I, collagenase IV, and DNase I (Solarbio, Beijing, China), following filtering through a 70 µm strainer to prepare single‐cell suspensions. Subsequently, the single‐cell suspensions were incubated with anti‐mouse CD16/CD32 antibody to block Fc receptors. To characterize the cell surface markers, blocked single‐cell suspensions were directly stained with the relevant antibodies for 30 min on ice and then washed twice in staining buffer. To characterize the intracellular markers, blocked cells were treated with Fixation/Permeabilization Solution Kit (BD, Franklin Lakes, NJ, USA) to break the cell membranes and then stained with PE anti‐human/mouse CD206. Flow cytometry analysis was performed using a CytoFLEX Flow Cytometer (Beckman Coulter, Brea, CA, USA), and the data were analyzed using FlowJo V10 (Tree Star Corp, Ashland, WI, USA). The antibody details are provided in Table S5 (Supporting Information).

### RNA Isolation and Quantitative Real‐Time PCR (RT‐qPCR)

Total RNA from cells and GC tissues was extracted using the RNA Quick Purification Kit (ES Science, Guangzhou, Guangdong, China) according to the manufacturer's protocols. Then, 1 µg of RNA was reverse‐transcribed to complementary DNA using *Evo M‐MLV* RT Kit (Accurate Biotechnology, Changsha, Hunan, China). Quantitative real‐time PCR was executed with SYBR Green Premix Pro Taq HS qPCR Kit (Accurate Biotechnology) on a LightCycler 480 system (Roche, Basel, Switzerland), following a two‐step RT‐qPCR protocol. Primer sequences are detailed in Table S4 (Supporting Information). Finally, gene expression levels were normalized to GAPDH and analyzed using the 2^−ΔΔCt^ method.

### Protein Extracting and Western Blotting (WB)

Total proteins from cells and tissues were extracted using pre‐cooled RIPA buffer (Beyotime) supplemented with phenylmethylsulfonyl fluoride and protease /phosphatase inhibitor cocktail (Beyotime). Nuclear and cytoplasmic proteins were isolated using the Nuclear and Cytoplasmic Protein Extraction Kit (Beyotime) according to the manufacturer's instructions. Protein concentrations were determined using the Omni‐Easy Instant BCA Protein Assay Kit (Epizyme Biotech, Shanghai, China). Equal amounts of protein lysates were separated using SDS‐PAGE and transferred to a PVDF membrane (Millipore, Billerica, MA, USA). Subsequently, the PVDF membranes were blocked with 5% skim milk at room temperature for 1.5 h and incubated with the corresponding primary antibody at 4 °C overnight. The next day, they were incubated with the corresponding secondary antibody at room temperature for 1 h, and the blot was visualized using ECL reagent (Millipore) and analyzed via ImageJ software. Antibodies information was detailed in Table S5 (Supporting Information).

### Co‐Immunoprecipitation (Co‐IP) and Mass Spectrometry Analysis

GC cells were lysed with Co‐IP lysis buffer (Beyotime) on ice for 30 min. Then, the cell lysates were clarified by centrifugation at 12000 rpm for 15 min at 4 °C. 10% cleared cell lysate was used for input. The rest of the cleared cell lysate was incubated with the specified antibody at 4 °C overnight. The next day, Protein A/G beads (Santa Cruz Biotechnology, Santa Cruz, CA, USA) were prewashed with lysis buffer solution and incubated with lysate‐antibody mixture for 4 h at 4 °C. Subsequently, the magnetic beads were extracted by the magnet holder and cleansed three times with a washing solution to remove unbound immune complexes. Bound immune complexes were resuspended in 1× SDS sample buffer and denatured at 97 °C for 7 min. Finally, western blot and mass spectrometry analyses were performed to analyze the protein components. Mass spectrometry analysis service was performed after Co‐IP to identify the LCN2‐binding proteins in GC cells with the assistance of Hui Jun Biological Technology Co., Ltd. (Guangzhou, Guangdong, China).

### Enzyme‐Linked Immunosorbent Assay (ELISA)

Supernatants from differently treated cells were collected and centrifuged at 1000 g for 20 min. The concentrations of CCL5, IL‐10, and VEGFC were determined using specific ELISA kits (Mlbio, Shanghai, China) according to the manufacturers' protocols. OD values at 450 nm were measured using a microplate reader (Bio‐TEK, Winooski, VT, USA) after adding the stop solution.

### Luminex Assay

Cytokine levels in different GC cell culture supernatants were measured by LabEx Co., Ltd. (Shanghai, China) using a Luminex assay. The expression levels of 46 cytokines were assessed using LX‐MultiDLH‐46 according to the manufacturer's instructions, and the results were read using the Luminex 200 System (LabEx).

### Luciferase Reporter Assay

NF‐κB luciferase reporter plasmid and pRL‐TK Renilla plasmid were acquired from Genechem Co., Ltd. (Shanghai, China) for luciferase assays. In brief, ≈1 × 10^6^ cells were seeded in 24‐well plates for 24 h before transfection with relevant plasmids using Lipofectamine 3000. After 48 h, luciferase activity was measured with a Dual‐Luciferase Reporter Assay Kit (Beyotime). The relative luciferase activity was expressed as the ratio of firefly to Renilla luciferase activity.

### Hematoxylin‐Eosin (HE) Staining and Immunohistochemistry (IHC)

Human and mice tumor tissues and corresponding LN tissues were collected, fixed, dehydrated, and embedded in paraffin for chopping into 4 µm slides. Subsequently, paraffin‐embedded sections were used for HE or IHC staining. HE and IHC staining were performed, and the IHC scores were calculated according to our previous study.^[^ ^31^ ^]^ Antibodies used for IHC were listed in Table S5 (Supporting Information).

### Immunofluorescence Staining Assay

For paraffin‐embedded sections, they first underwent a series of steps including deparaffinization, rehydration, and antigen retrieval. For cells, 5 × 10^3^ cells were seeded on glass coverslips in 24‐well plates. After culturing for 24 h at 37 °C, cells on slides were fixed with 4% paraformaldehyde for 15 min and then permeabilized with 0.25% Triton X‐100 (Solarbio) at room temperature for 20 min. Subsequently, the sections and fixed cells were blocked with 20% goat serum at room temperature for 30 min and followed by incubation in the corresponding primary antibody at 4 °C overnight. The next day, after washing three times with PBST, fluorescent secondary antibody (Invitrogen) was added and incubated at room temperature for 2 h in the dark. Finally, the cell nuclei were stained with 4′,6‐diamidino‐2‐phenylindole (DAPI; Solarbio) for 5–10 min, and images of the sections and cells were captured using a fluorescence microscope (Leica, Wetzlar, Germany).

### HLECs Tube Formation and Transwell Assays

FBS‐free culture medium supernatant obtained from different groups was concentrated using ultrafiltration spin columns. Aliquots (300 µL) of a mixture of growth factor‐reduced Matrigel (Corning) and serum‐free ECM (1:2, v/v) were pre‐coated into each well of a pre‐cooled 24‐well plate and incubated at 37 °C for 4 h. 3 × 10^4^ HLECs/MLECs were then seeded into the 24‐well plate containing 500 µL of the respective culture medium supernatant and incubated for 8 h. Images of lymphatic tubes were captured using an inverted microscope and the length of the tubes was quantified using Image J software. Transwell assays were performed to evaluate the migration ability of HLECs. In brief, HLECs, after treatment with different culture medium supernatants, were harvested and resuspended in serum‐free ECM. Then, 1 × 10^5^ treated HLECs in a total volume of 300 µL of serum‐free medium were placed in the upper chamber of a transwell assay (24‐well chambers, 8.0 µm pore membrane; Corning), whereas the lower chambers were filled with 600 µL of the medium containing 10% FBS. After 16 h incubation, the migrated cells were fixed in 4% paraformaldehyde for 30 min and then stained with crystal violet for 20 min. Images were captured using an inverted microscope, and the migrated cells in six randomly selected fields were counted using Image J software.

### Statistical Analysis

Statistical analyses were performed via GraphPad Prism 9.0 (GraphPad Software, San Diego, CA, USA). Experimental data were expressed as mean ± SD from at least three independent duplications. Statistical tests, including Student's *t* test, one‐way or two‐way ANOVA, Mann‐Whitney U test, and Kruskal–Wallis test, were applied appropriately to compare group differences based on the experimental design and data outcomes. The log‐rank test was applied to examine survival differences using the Kaplan‐Meier method. Significance level was defined at ^*^ *p* < 0.05, ^**^ *p *< 0.01, ^***^ *p *< 0.001, and ^****^ *p *< 0.0001.

### Ethics Approval Statement

This study was approved by the Institutional Ethical Boards of the First Affiliated Hospital of Sun Yat‐sen University (project number: 2020–164). Written informed consent was obtained from all patients. The animal study was carried out in compliance with the guidance of the Animal Care Committee of the First Affiliated Hospital of Sun Yat‐sen University (approval number: [2025]030).

## Conflict of Interest

The authors declare no conflict of interest.

## Author Contributions

Z.H., Y.L., Y.Q., L.Y. contributed equally to this work. Z.H. contributed to investigation, methodology, formal analysis, data curation, conceptualization, wrote the original draft, wrote, reviewed & edited the final manuscript. Y.L. contributed to data curation, wrote the original draft, wrote, reviewed & edited the final manuscript. Y.Q. contributed to formal analysis and data curation, acquired funding and wrote, reviewed & edited the final manuscript. L.Y. wrote, reviewed & edited the final manuscript, contributed to methodology and formal analysis. T.Z. contributed to methodology and formal analysis, acquired funding. Y.C. contributed to methodology and formal analysis. J.W. wrote, reviewed & edited the final manuscript, and contributed to data curation. P.D. contributed to methodology and formal analysis. T.Z. contributed to investigation and data curation. Z.Y. contributed to investigation and data curation. Z.Z. contributed to formal analysis and data curation. R.Z. contributed to methodology, data curation, and acquired funding. Z.L. contributed to formal analysis and worked with the software. E.Z. contributed to validation and project administration, supervised the project, and acquired resources. S.C. and J.C. contributed to validation and project administration, supervised the project, and acquired resources and funding.

## Supporting information



Supporting Information

Supplemental Table 7

Supplemental Table 8

Supplemental Table 9

Supplemental Table 10

Supplemental Table 11

Supplemental Table 12

## Data Availability

The data that support the findings of this study are available from the corresponding author upon reasonable request.

## References

[1] H. Sung , J. Ferlay , R. L. Siegel , M. Laversanne , I. Soerjomataram , A. Jemal , F. Bray , Ca‐Cancer J. Clin. 2021, 71, 209. 33538338 10.3322/caac.21660

[2] E. C. Smyth , M. Nilsson , H. I. Grabsch , N. C. van Grieken , F. Lordick , Lancet 2020, 396, 635. 32861308 10.1016/S0140-6736(20)31288-5

[3] M. A. Shah , J. Clin. Oncol. 2015, 33, 1760. 25918288 10.1200/JCO.2014.60.1799

[4] Japanese Gastric Cancer Association , Gastric Cancer 2021, 24, 1. 11957040 10.1007/s101209800016

[5] M. M. Abdelfatah , M. Barakat , H. Lee , J. J. Kim , N. Uedo , I. Grimm , M. O. Othman , Gastrointest. Endosc. 2018, 87, 338. 28966062 10.1016/j.gie.2017.09.025

[6] H. Khalayleh , Y.‐W. Kim , H. M. Yoon , K. W. Ryu , JAMA Network Open 2021, 4, 211840. 10.1001/jamanetworkopen.2021.1840 PMC7970333 33729506

[7] E. L. Vos , M. Nakauchi , M. Gönen , J. A. Castellanos , A. Biondi , D. G. Coit , J. L. Dikken , D. D'ugo , H. Hartgrink , P. Li , M. Nishimura , M. Schattner , K. Y. Song , L. H. Tang , I. Uyama , S. Vardhana , R. H. Verhoeven , B. P. Wijnhoven , V. E. Strong , Ann. Surg. 2023, 277, 339. 10.1097/SLA.0000000000005332 PMC9192823 34913904

[8] S. A. Stacker , S. P. Williams , T. Karnezis , R. Shayan , S. B. Fox , M. G. Achen , Nat. Rev. Cancer 2014, 14, 159. 24561443 10.1038/nrc3677

[9] T. Tammela , K. Alitalo , Cell 2010, 140, 460. 20178740 10.1016/j.cell.2010.01.045

[10] S. Li , Q. Li , Cancer Letters 2015, 357, 438. 25497008 10.1016/j.canlet.2014.12.013

[11] W. Li , J. M. Gauthier , A. Y. Tong , Y. Terada , R. Higashikubo , C. C. Frye , M. S. Harrison , K. Hashimoto , A. I. Bery , J. H. Ritter , R. G. Nava , V. Puri , B. W. Wong , K. J. Lavine , A. Bharat , A. S. Krupnick , A. E. Gelman , D. Kreisel , J. Clin. Invest. 2020, 130, 6718. 33196461 10.1172/JCI136057 PMC7685742

[12] Y. Yang , Y. Cao , Semin. Cancer Biol. 2022, 86, 251. 35307547 10.1016/j.semcancer.2022.03.011

[13] M. Skobe , T. Hawighorst , D. G. Jackson , R. Prevo , L. Janes , P. Velasco , L. Riccardi , K. Alitalo , K. Claffey , M. Detmar , Nat. Med. 2001, 7, 192. 11175850 10.1038/84643

[14] E. A. Korhonen , A. Murtomäki , S. K. Jha , A. Anisimov , A. Pink , Y. Zhang , S. Stritt , I. Liaqat , L. Stanczuk , L. Alderfer , Z. Sun , E. Kapiainen , A. Singh , I. Sultan , A. Lantta , V.‐M. Leppänen , L. Eklund , Y. He , H. G. Augustin , K. Vaahtomeri , P. Saharinen , T. Mäkinen , K. Alitalo , J. Clin. Invest. 2022, 132, 155478. 10.1172/JCI155478 PMC9337826 35763346

[15] H. Ji , R. Cao , Y. Yang , Y. Zhang , H. Iwamoto , S. Lim , M. Nakamura , P. Andersson , J. Wang , Y. Sun , S. Dissing , X. He , X. Yang , Y. Cao , Nat. Commun. 2014, 5, 4944. 25229256 10.1038/ncomms5944

[16] Q. Zhang , S. Liu , H. Wang , K. Xiao , J. Lu , S. Chen , M. Huang , R. Xie , T. Lin , X. Chen , Adv. Sci. 2023, 10, 2205613. 10.1002/advs.202205613 PMC10104629 36670069

[17] J. Song , W. Chen , X. Cui , Z. Huang , D. Wen , Y. Yang , W. Yu , L. Cui , C.‐Y. Liu , Theranostics 2020, 10, 2327. 32089745 10.7150/thno.39740 PMC7019157

[18] G. Marone , G. Varricchi , S. Loffredo , F. Granata , Eur. J. Pharmacol. 2016, 77, 146. 10.1016/j.ejphar.2015.03.088 25941082

[19] M. Bied , W. W. Ho , F. Ginhoux , C. Blériot , Cellul. Molecul. Immunol. 2023, 20, 983. 10.1038/s41423-023-01061-6 PMC10468537 37429944

[20] Y. Lin , J. Xu , H. Lan , Journal of Hematology and Oncology 2019, 12, 76. 31300030 10.1186/s13045-019-0760-3 PMC6626377

[21] V. Gambardella , J. Castillo , N. Tarazona , F. Gimeno‐Valiente , C. Martínez‐Ciarpaglini , M. Cabeza‐Segura , S. Roselló , D. Roda , M. Huerta , A. Cervantes , T. Fleitas , Cancer Treat. Rev. 2020, 86, 102015. 32248000 10.1016/j.ctrv.2020.102015

[22] R. P. Kataru , K. Jung , C. Jang , H. Yang , R. A. Schwendener , J. E. Baik , S. H. Han , K. Alitalo , G. Y. Koh , Blood 2009, 113, 5650. 19346498 10.1182/blood-2008-09-176776

[23] C. Chen , W. He , J. Huang , B. Wang , H. Li , Q. Cai , F. Su , J. Bi , H. Liu , B. Zhang , N. Jiang , G. Zhong , Y. Zhao , W. Dong , T. Lin , Nat. Commun. 2018, 9, 3826. 30237493 10.1038/s41467-018-06152-x PMC6148066

[24] J. J. Rodvold , N. R. Mahadevan , M. Zanetti , Cancer Letters 2012, 316, 132. 22075378 10.1016/j.canlet.2011.11.002

[25] P. Chandrasekaran , S. Weiskirchen , R. Weiskirchen , Int. J. Mol. Sci. 2024, 25, 4290.38673873

[26] M. Feng , J. Feng , W. Chen , W. Wang , X. Wu , J. Zhang , F. Xu , M. Lai , Molecular Cancer 2016, 15, 77. 27912767 10.1186/s12943-016-0564-9 PMC5135816

[27] C. Shi , C. Wang , Z. Fu , J. Liu , Y. Zhou , B. Cheng , C. Zhang , S. Li , Y. Zhang , Pharmacological Research 2024, 201, 107088. 38295916 10.1016/j.phrs.2024.107088

[28] S. Nishimura , Y. Yamamoto , A. Sugimoto , S. Kushiyama , S. Togano , K. Kuroda , T. Okuno , H. Kasashima , M. Ohira , K. Maeda , M. Yashiro , Gastric Cancer 2022, 25, 850. 35705840 10.1007/s10120-022-01305-w PMC9365736

[29] S. A. Koh , K. H. Lee , Oncol. Rep. 2015, 34, 2179. 26259977 10.3892/or.2015.4189

[30] J. Xu , S. Lv , W. Meng , F. Zuo , Cancer Manage. Res. 2020, 12, 12841. 10.2147/CMAR.S278902 PMC7751782 33364832

[31] Z. Huang , Y. Li , Y. Qian , E. Zhai , Z. Zhao , T. Zhang , Y. Liu , L. Ye , R. Wei , R. Zhao , Z. Li , Z. Liang , S. Cai , J. Chen , Cell Death Dis. 2024, 15, 756. 39424639 10.1038/s41419-024-07153-z PMC11489581

[32] Y. Qian , E. Zhai , S. Chen , Y. Liu , Y. Ma , J. Chen , J. Liu , C. Qin , Q. Cao , J. Chen , S. Cai , Int. J. Cancer 2022, 151, 1367. 35716132 10.1002/ijc.34172

[33] C. T. Kureshi , S. K. Dougan , Cancer Cell 2025, 43, 15. 39672170 10.1016/j.ccell.2024.11.011 PMC11841838

[34] L. Gu , Y. Sang , X. Nan , Y. Zheng , F. Liu , L. Meng , M. Sang , B. Shan , Molecular Cancer 2022, 21, 217. 36514094 10.1186/s12943-022-01686-7 PMC9746112

[35] F. M. Farina , S. Serio , I. F. Hall , S. Zani , G. A. Cassanmagnago , M. Climent , E. Civilini , G. Condorelli , M. Quintavalle , L. Elia , Eur. Heart J. 2022, 43, 4562. 35292818 10.1093/eurheartj/ehac097

[36] X. Kuang , Z. Zhang , D. Li , W. Bao , J. Pan , P. Zhou , H. Chen , Z. Gao , X. Xie , C. Yang , G. Zhu , Z. Zhou , R. Tang , Z. Feng , L. Zhou , X. Feng , L. Wang , J. Yang , L. Jiang , Cellul. Molecul. Biol. Letters 2023, 28, 62. 10.1186/s11658-023-00465-6 PMC10388466 37525118

[37] H. Chen , X. Chen , Z. Zhang , W. Bao , Z. Gao , D. Li , X. Xie , P. Zhou , C. Yang , Z. Zhou , J. Pan , X. Kuang , R. Tang , Z. Feng , L. Zhou , D. Zhu , J. Yang , L. Wang , H. Huang , D. Tang , J. Liu , L. Jiang , Oncogene 2022, 41, 5253. 36316443 10.1038/s41388-022-02520-6

[38] S. Karaman , M. Detmar , J. Clin. Invest. 2014, 124, 922. 24590277 10.1172/JCI71606 PMC3938272

[39] S. Zhao , Y. Mi , B. Guan , B. Zheng , P. Wei , Y. Gu , Z. Zhang , S. Cai , Y. Xu , X. Li , X. He , X. Zhong , G. Li , Z. Chen , D. Li , J. Hematol. Oncol. 2020, 13, 156. 33213490 10.1186/s13045-020-00991-2 PMC7678301

[40] L. Song , X. Yu , Y. Wu , W. Zhang , Y. Zhang , Y. Shao , Z. Hou , C. Yang , Y. Gao , Y. Zhao , Adv. Sci. 2025, 12, 2406865. 10.1002/advs.202406865 PMC11727125 39535362

[41] Y. He , D. Feng , S. Hwang , B. Mackowiak , X. Wang , X. Xiang , R. M. Rodrigues , Y. Fu , J. Ma , T. Ren , Y. Ait‐Ahmed , M. Xu , S. Liangpunsakul , B. Gao , J. Hepatol. 2021, 75, 163. 33610678 10.1016/j.jhep.2021.02.004 PMC8323118

[42] W. Ouyang , A. O'Garra , Immunity 2019, 50, 871. 30995504 10.1016/j.immuni.2019.03.020

[43] J.‐Y. Deng , H. Liang , World J. Gastroenterol. 2014, 20, 3967. 24744586 10.3748/wjg.v20.i14.3967 PMC3983452

[44] S. A. Jaberi , A. Cohen , C. D'Souza , Y. M. Abdulrazzaq , S. Ojha , S. Bastaki , E. A. Adeghate , Biomedic. Pharmacother. 2021, 142, 112002. 10.1016/j.biopha.2021.112002 34463264

[45] H. Wang , X. Wang , X. Zhang , W. Xu , Drug Resist. Updates 2024, 7, 101041. 10.1016/j.drup.2023.101041 38198845

[46] H. Zheng , C. Chen , Y. Luo , M. Yu , W. He , M. An , B. Gao , Y. Kong , Y. Ya , Y. Lin , Y. Li , K. Xie , J. Huang , T. Lin , Clin. Transl. Med. 2021, 11, 497. 10.1002/ctm2.497 PMC8288020 34323412

[47] M. J. Karkkainen , P. Haiko , K. Sainio , J. Partanen , J. Taipale , T. V. Petrova , M. Jeltsch , D. G. Jackson , M. Talikka , H. Rauvala , C. Betsholtz , K. Alitalo , Nat. Immunol. 2004, 5, 74. 14634646 10.1038/ni1013

[48] L. F. Shi , Y. Wu , C. Y. Li , J. Gynecolog. Oncol. 2015, 26, 327. 10.3802/jgo.2015.26.4.327 PMC4620370 26197772

[49] B. Pytowski , J. Goldman , K. Persaud , Y. Wu , L. Witte , D. J. Hicklin , M. Skobe , K. C. Boardman , M. A. Swartz , J. Nation. Cancer Instit. 2005, 97, 14. 10.1093/jnci/dji003 15632376

[50] D. Aldinucci , N. Casagrande , Int. J. Mol. Sci. 2018, 19, 1477. 29772686 10.3390/ijms19051477 PMC5983686

[51] J. Xu , Q. Shi , J. Lou , B. Wang , W. Wang , J. Niu , L. Guo , C. Chen , Y. Yu , Y. Huang , W. Guo , J. Lan , Y. Zhu , T. Ren , X. Tang , J. Immuno. Ther. Cancer 2023, 11, 006808. 10.1136/jitc-2023-006808 PMC10151997 37185233

[52] Y. Nie , H. Huang , M. Guo , J. Chen , W. Wu , W. Li , X. Xu , X. Lin , W. Fu , Y. Yao , F. Zheng , M.‐L. Luo , P. E. Saw , H. Yao , E. Song , H. Hu , Clin. Cancer Res. 2019, 25, 3873. 30890553 10.1158/1078-0432.CCR-18-3421

[53] Z. Zeng , T. Lan , Y. Wei , X. Wei , Genes and Diseases 2022, 9, 12. 34514075 10.1016/j.gendis.2021.08.004 PMC8423937

[54] M. A. Salkeni , A. Naing , Trends Cancer 2023, 9, 716. 37321942 10.1016/j.trecan.2023.05.003 PMC10524969

