# Supplementary material for: An LCN2‐Dependent Positive‐Feedback Loop Between Gastric Cancer Cells and Tumor‐Associated‐Macrophages Mediates Lymphangiogenesis and Lymphatic Metastasis
Source: Adv Sci (Weinh). 2025 Aug 30;12(44):e08352. doi: 10.1002/advs.202508352 (PMC12667518; doi:10.1002/advs.202508352)
Supplement: Supplementary file 1 — Supporting Information [file ADVS-12-e08352-s002.docx]

**Supporting Information**

**Title: An LCN2-Dependent Positive-Feedback Loop Between Gastric Cancer Cells and Tumor-Associated-Macrophages Mediates Lymphangiogenesis and Lymphatic Metastasis**

**Authors:** *Zhixin Huang*^1,2,#^, *Ying Li*^3,#^, *Yan Qian*^1,#^, *Linying Ye*^1,2,#^, *Tianhao Zhang*^1,2^, *Yang Cheng*^1,2^, *Jialin Wu*^3^, *Peng Duan*^1,2^, *Tiantian Zhang*^3^, *Zihan Yu*^1,2^, *Zeyu Zhao*^1,2^, *Risheng Zhao*^1^, *Zhi Liang*^1,2^, *Ertao Zhai*^1,*^, *Shirong Cai*^1,*^, *Jianhui Chen*^1,4,*^

**Affiliations:** ^1^Division of Gastrointestinal Surgery Center, the First Affiliated Hospital of Sun Yat-sen University, Guangzhou, 510080, Guangdong, China

^2^Laboratory of Surgery, the First Affiliated Hospital of Sun Yat-sen University, Guangzhou, 510080, Guangdong, China

^3^Guangdong Provincial Key Laboratory of Microbial Safety and Health, State Key Laboratory of Applied Microbiology Southern China, Institute of Microbiology, Guangdong Academy of Sciences, Guangzhou, 510070, Guangdong, China

^4^Department of General Surgery, Guangxi Hospital Division of The First Affiliated Hospital, Sun Yat-sen University, Nanning 530000 Guangxi, China.

^#^These authors contributed equally to this work.

**Corresponding author:**

Ertao Zhai, Division of Gastrointestinal Surgery Center, the First Affiliated Hospital of Sun Yat-sen University, Guangzhou, 510080, Guangdong, China

Email: zhaiert@mail2.sysu.edu.cn

Shirong Cai, Division of Gastrointestinal Surgery Center, the First Affiliated Hospital of Sun Yat-sen University, Guangzhou, 510080, Guangdong, China

Email: caishr@mail.sysu.edu.cn

Jianhui Chen, Division of Gastrointestinal Surgery Center, the First Affiliated Hospital of Sun Yat-sen University, Guangzhou, 510080, Guangdong, China

Email: chenjh45@mail.sysu.edu.cn

**Supplementary Figures**


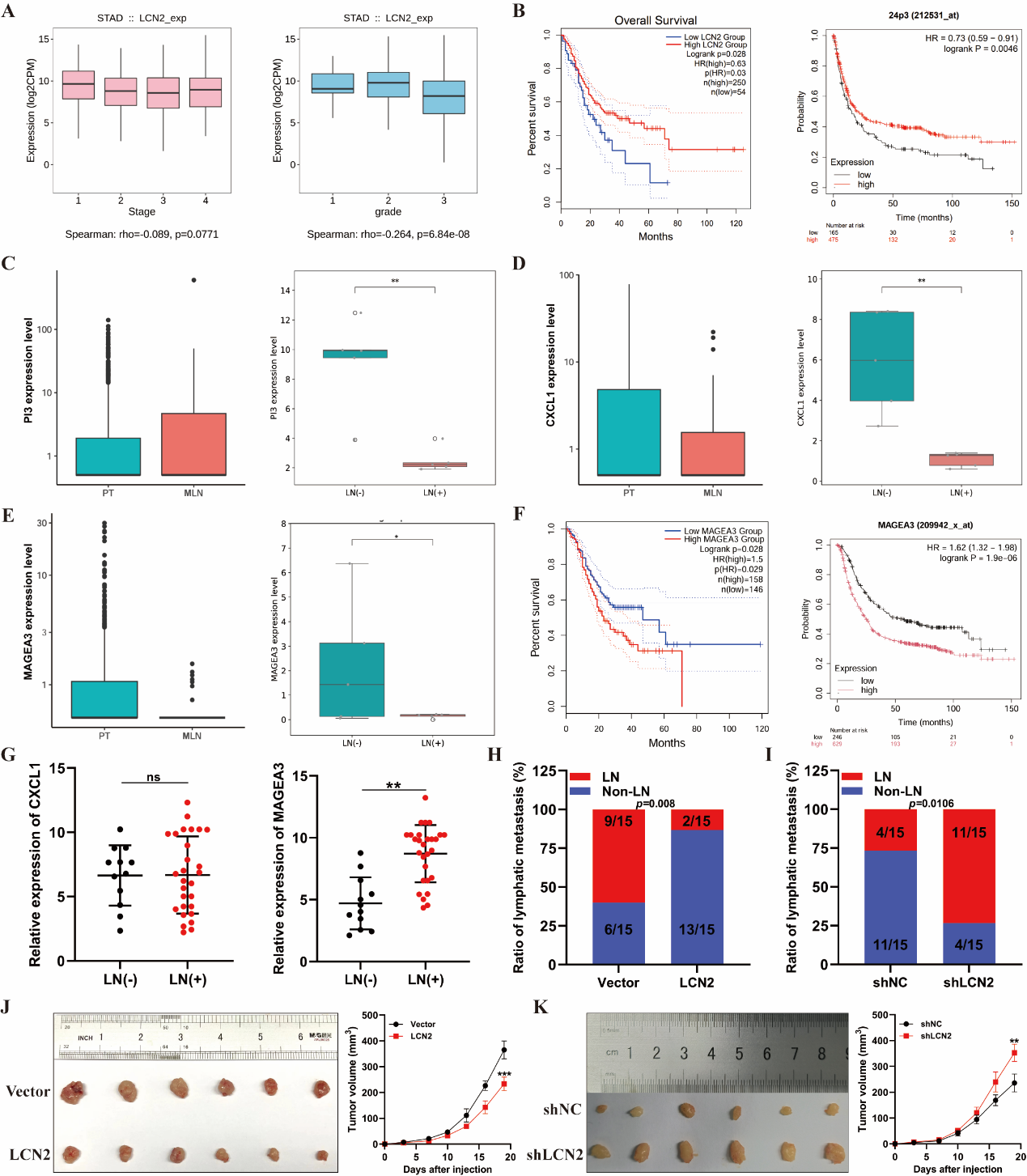


**Supplementary Figure S1. Lowered‌ LCN2 is associated with increased lymph-node metastasis and shorter survival in GC**

**A** Correlation between LCN2 expression in gastric cancer tissues from TCGA-STAD and pathological grade and stage of patients with gastric cancer.

**B** Kaplan-Meier survival analysis of the OS for patients with GC with low versus high LCN2 expression in TCGA and KM-plot database.

**C-E** The expression levels of PI3, CXCL1, and MAGEA3 in primary tumors (PT) and metastatic lymph nodes (MLN) of gastric cancer from our previous single-cell RNA sequencing data and in 5 LN-metastasis-negative LN(−) and 5 LN-metastasis-positive LN(+) primary gastric cancer tissue from RNA sequencing data.

**F** Kaplan-Meier survival analysis of the OS for patients with GC with low versus high MAGEA3 expression in TCGA and KM-plot database.

**G** The mRNA expression of CXCL1 and MAGEA3 in freshly collected gastric cancer tissues with (n=28) or without (n=12) LN metastasis.

**H, I** Ratios of the number of metastatic and non- metastatic to total enucleated popliteal LNs for the indicated groups.

**J, K** Images of xenograft tumor samples (left) and tumor volumes (right).

Data are expressed as mean ± SD of biological replicate experiments. * indicates *p* < 0.05, ** indicates *p* < 0.01, and ns indicates no significance.


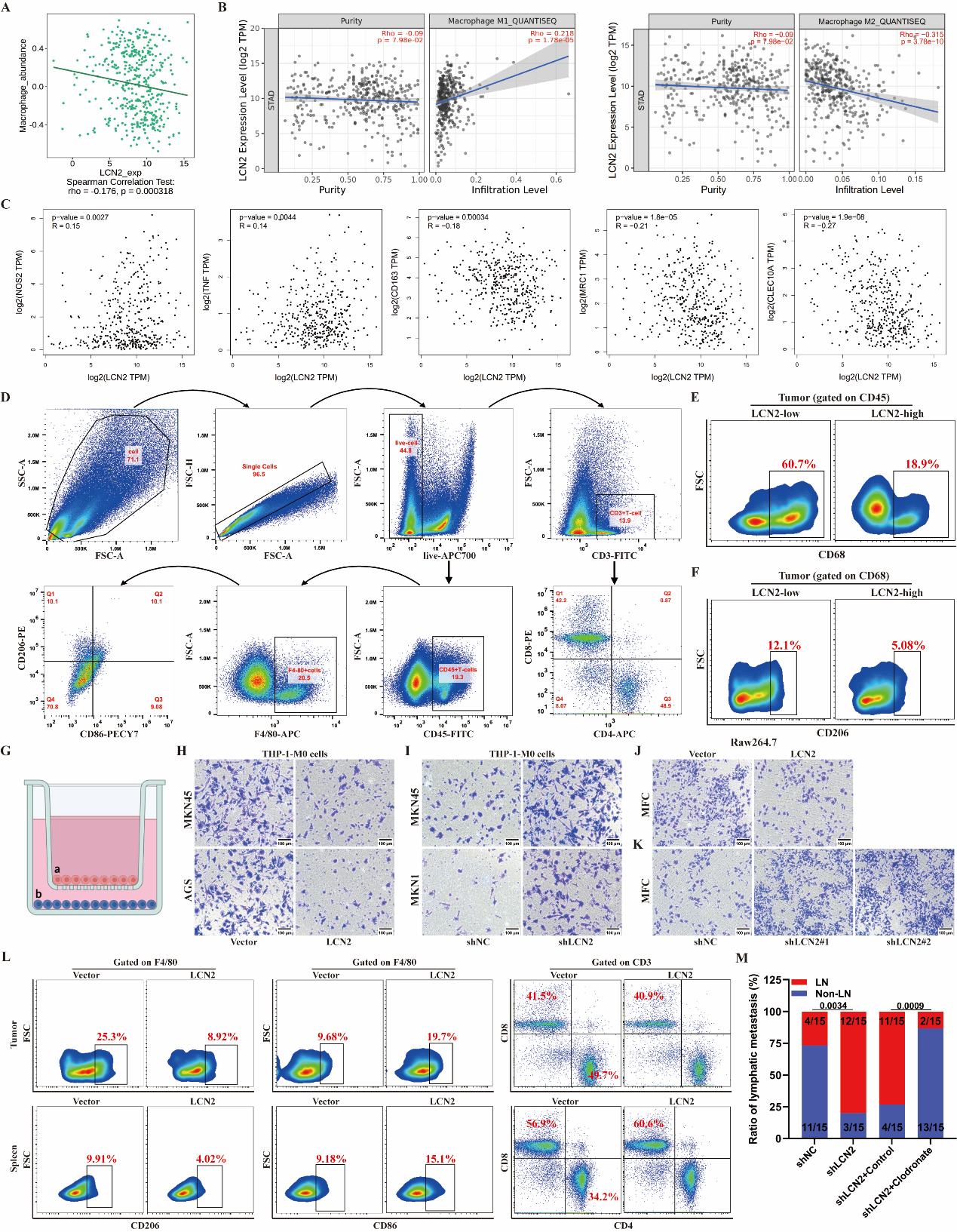


**Supplementary Figure S2. Tumor-associated macrophages are integral for LCN2-mediated inhibition of lymph-node metastasis in GC**

**A** Correlation between LCN2 expression and macrophage abundance among gastric cancer tissues from TCGA-STAD via TISIDB.

**B** Correlation between LCN2 expression and the infiltration of M1-type macrophages and M2-type macrophages among gastric cancer tissues from TCGA-STAD via TIMER2.0 by QUANSTISED.

**C** Correlation between LCN2 expression and M1-type TAM markers (NOS2, TNF-α) and M2-type TAM markers (CD163, CD206, CD301) expression among gastric cancer tissues from TCGA-STAD.

**D** The gating strategies used for cell surface markers analysis.

**E, F** Representative flow cytometry images for CD45^+^CD68^+^ macrophages (E) and CD206^+^ TAMs (F) in fresh surgically excised human GC tissues with different LCN2 expression levels.

**G** Schematic of in vitro model of co-culture and transwell assay between macrophages and GC cells.

**H-K** Representative images of transwell assay in THP-1-derived M0 macrophages and Raw264.7 cells. Transwell assays were used to assess the effect of conditioned medium collected from the indicated human GC cells on the chemotactic ability of THP-1-derived M0 macrophages.

**L** Representative flow cytometric images for CD45^+^CD68^+^ macrophages (E) and CD206^+^ TAMs (F) in fresh surgically excised human GC tissues with different LCN2 expression levels.

**M** Ratios of the number of metastatic and non-metastatic to total enucleated popliteal LNs for the indicated groups.


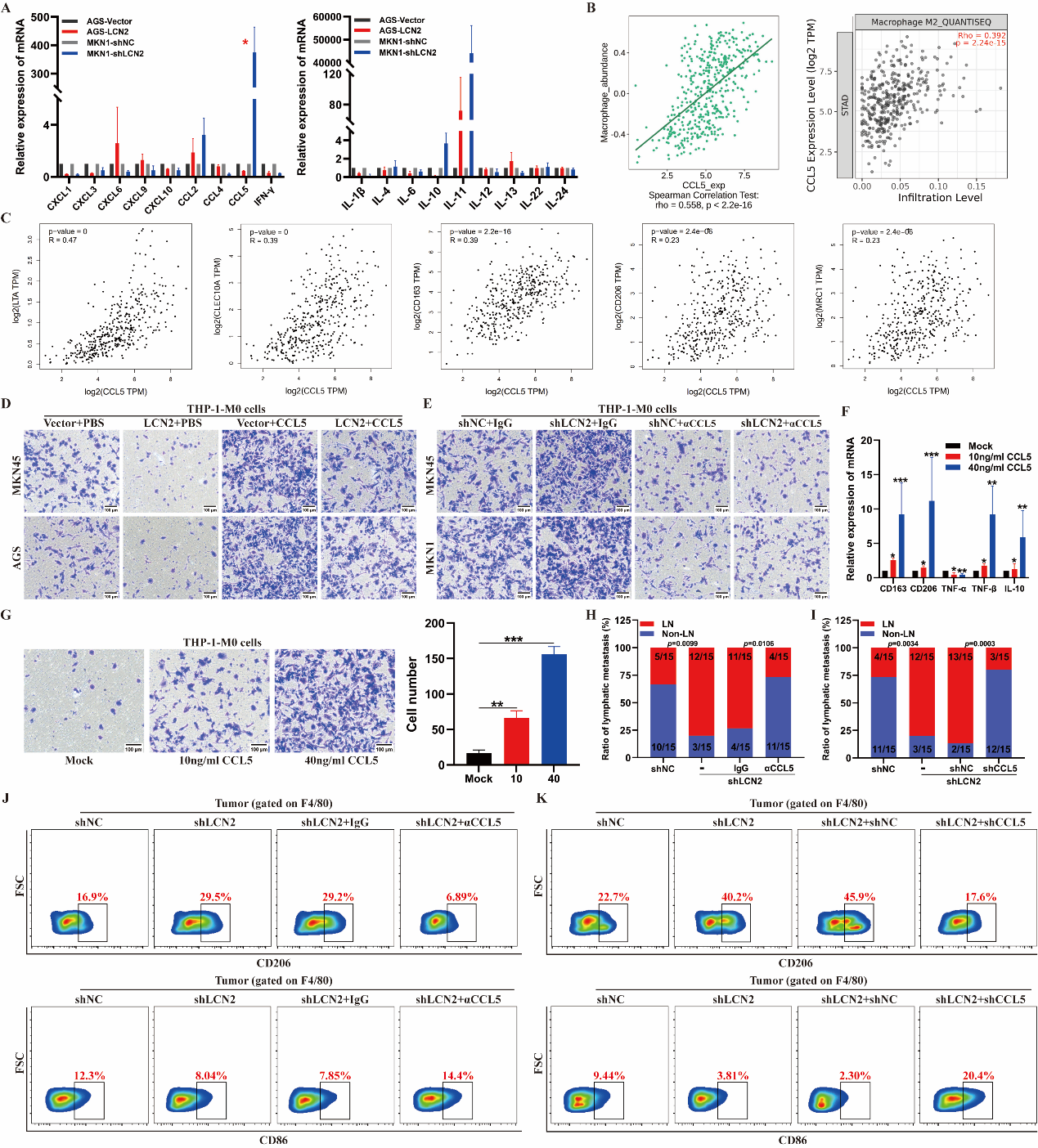


**Supplementary Figure S3. CCL5 is essential for LCN2-mediated inhibition of macrophages activation and lymph-node metastasis in GC**

**A** RT-qPCR analyses of the corresponding cytokines and chemokines expression in LCN2-overexpression, LCN2-silenced and control GC cells as indicated.

**B** Correlation between CCL5 expression and macrophage abundance among gastric cancer tissues from TCGA-STAD.

**C** Correlation between CCL5 expression and M1-type TAM markers and M2-type TAM markers expression among gastric cancer tissues from TCGA-STAD.

**D, E** Representative images of transwell assay in THP-1-derived M0 macrophages with corresponding treatment.

**F** The expression of biomarkers of M1-like and M2-like macrophages in THP-1-derived M0 macrophages after treating with different concentrations of CCL5.

**G** Representative images (left) and quantitative results (right) of transwell assay in THP-1-derived M0 macrophages treating with different concentrations of CCL5.

**H, I** Ratios of the number of metastatic and non-metastatic to total enucleated popliteal LNs for the indicated groups.

**J, K** Representative flow cytometric images for CD45^+^F4/80^+^CD206^+^ cells and CD45^+^F4/80^+^CD86^+^ cells in mice tumor tissues from the indicated groups

Data are expressed as mean ± SD of biological replicate experiments. * indicates *p* < 0.05, ** indicates *p* < 0.01, and *** indicates *p* < 0.001

**
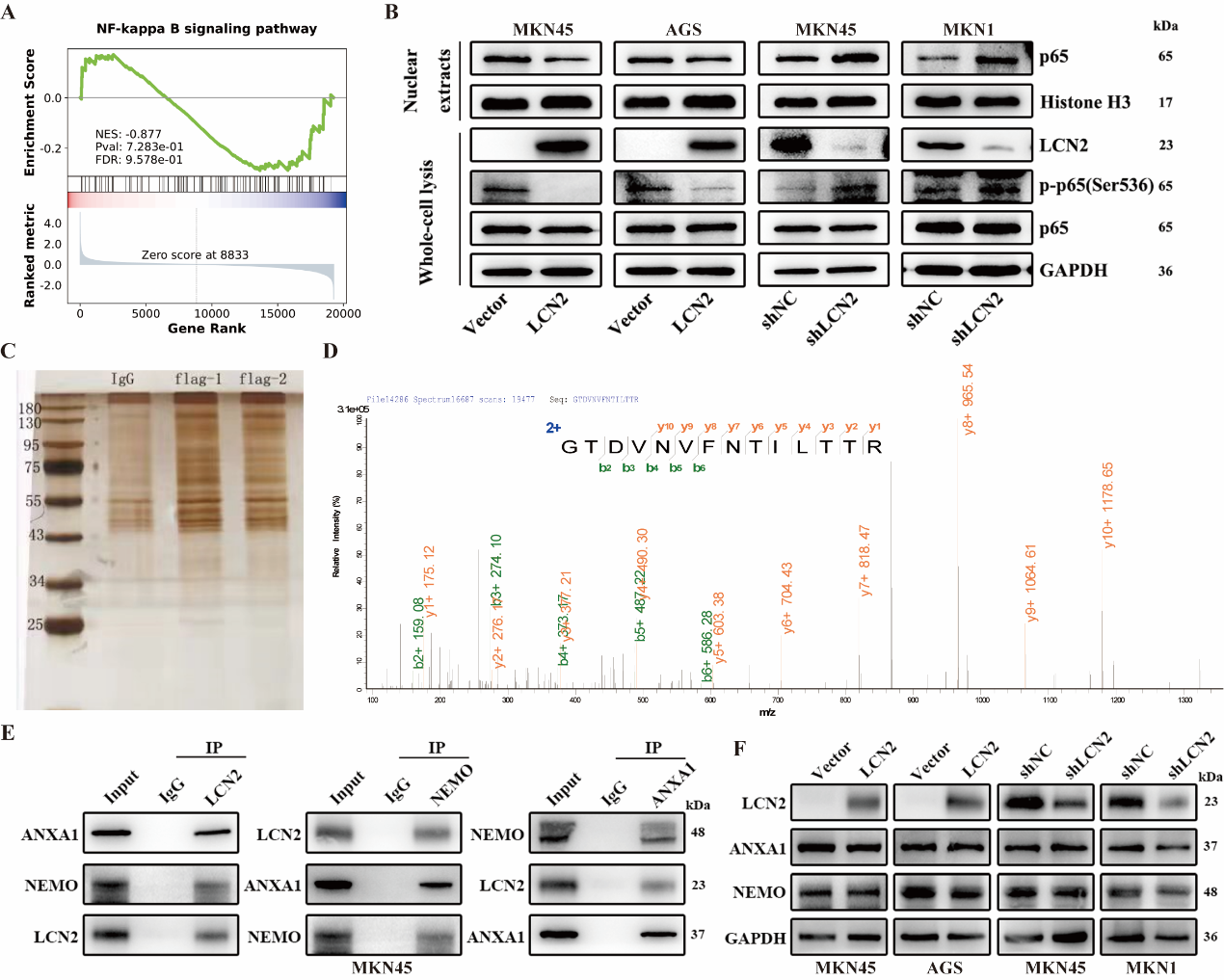
**

**Supplementary Figure S4. LCN2 downregulates CCL5 expression by inhibiting the activation of the NF-κB pathway via ANXA1-dependent poly-ubiquitination of NEMO**

**A** GSEA revealed that LCN2 is negatively associated with the NF-κB activation.

**B** Western blotting analysis of subcellular localization of NF-κB/p65 in the indicated cells. Histone H3 was used as a nuclear loading control. GAPDH was used as a cytoplasmic loading control.

**C D** Lysates from AGS cells transfected with LCN2-overexpression plasmid were immunoprecipitated with anti-LCN2 antibody. Silver stain and the mass spectrometry peptide sequencing revealed that there was the interaction between LCN2 and ANXA1.

**E** The lysates of MKN45 cells transfected with LCN2-overexpression plasmid were immunoprecipitated with antibodies against LCN2, ANXA1 and NEMO.

**F** Western blot analysis of the effect of LCN2 on ANXA1 and NEMO expression in the indicated GC cells.

**
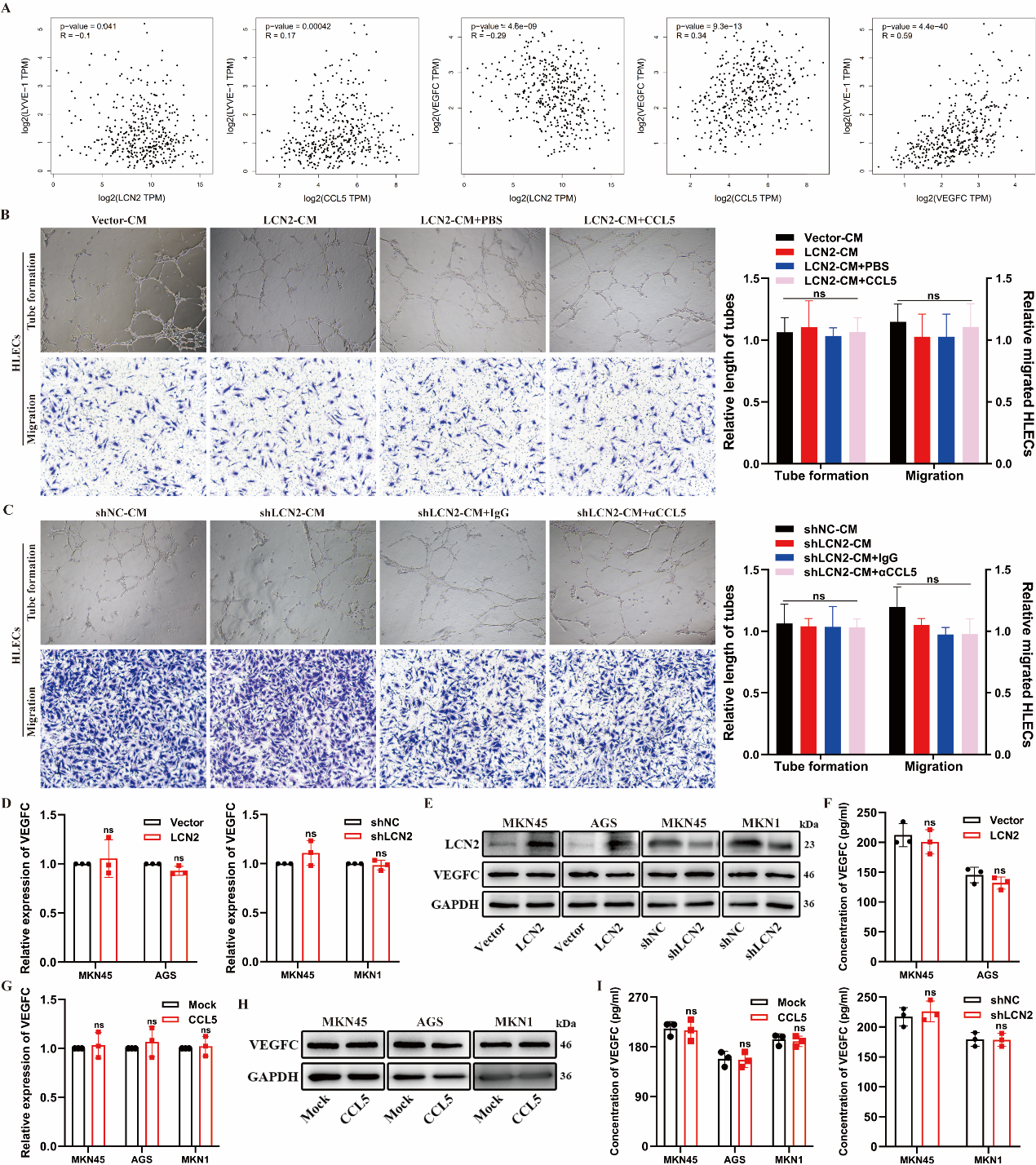
**

**Supplementary Figure S5. CCL5-activated TAMs induce lymphangiogenesis and lymph-node metastasis through the VEGFC/VEGFR3 pathway**

**A** Correlation analysis of LCN2, CCL5, VEGFC, and LYVE-1 expression in gastric cancer tissues from TCGA-STAD via GEPIA2.

**B, C** Representative images (left) and quantification (right) of tube formation and transwell invasion by HLECs treated with the conditioned medium collected from gastric cancer cells with the corresponding treatment.

**D-F** RT-qPCR (B), western blot (C), and ELISA (D) analyses of VEGFC expression in LCN2-overexpressing (LCN2), LCN2-silenced (shLCN2), and control GC cells.

**G-I** RT-qPCR (E), western blot (F), and ELISA (G) analyses of VEGFC expression in control GC cells and CCL5-treated GC cells.

Data are expressed as mean ± SD of biological replicate experiments. ns indicates no significance.


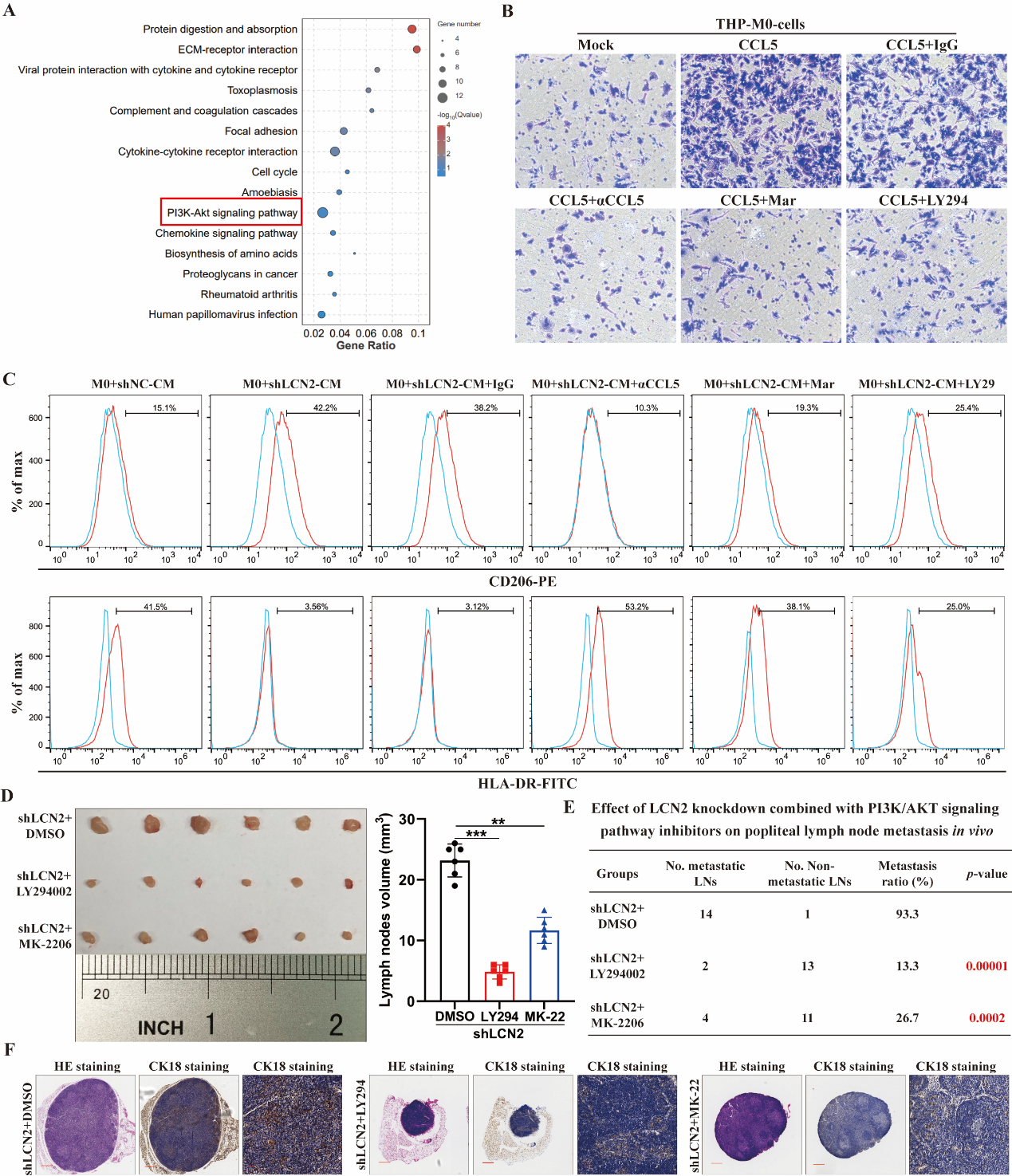


**Supplementary Figure S6. A positive-feedback loop of the CCL5/CCR5/PI3K/AKT/GSK3β/IL-10/IκBζ/LCN2 axis is formed between gastric cancer cells and tumor associated-macrophages.**

**A** KEGG pathway enrichment analysis of differentially expressed genes between PBS- and CCL5- treated THP-1-derived M0 macrophages.

**B** Representative images (left) of transwell assay in THP-1-derived M0 macrophages with corresponding treatment. THP-1-derived M0 macrophages were firstly treated with conditioned medium collected from the indicated GC cells for 24 h, and then CCL5-neutralizing antibody, CCR5 inhibitor, and PI3K inhibitor treatment was carried out for an additional 12 h.

**C** Representative flow cytometric images for CD206^+^ cells and HLA-DR^+^ cells in THP-1-derived M0 macrophages with corresponding treatment.

**D** Representative images (left) and volumes (right) of the enucleated popliteal LNs in the indicated mouse groups.

**E** Ratios of metastatic and non-metastatic enucleated popliteal LNs to the total count for the indicated groups.

**F** Representative images of HE and CK-18 IHC staining of the popliteal lymph nodes for the indicated groups.

Data are expressed as the mean ± SD of biological replicate experiments. * indicates *p* < 0.05, ** indicates *p* < 0.01, *** indicates *p* < 0.001, **** indicates *p* < 0.0001, and ns indicates no significance.


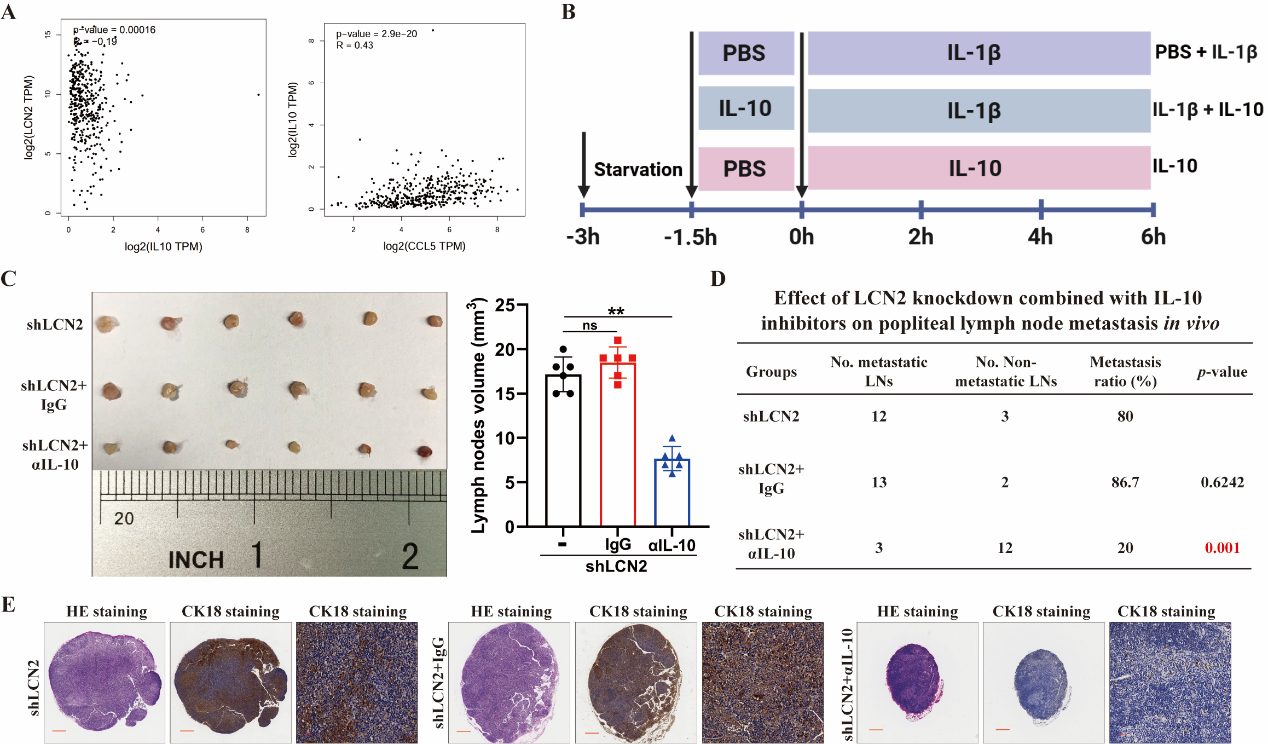


**Supplementary Figure S7. TAMs-derived IL-10 inhibits the expression of** **IκBζ and its target gene LCN2 in GC cells by promoting IκBζ degradation**

**A** Correlation analysis between IL-10 and LCN2, CCL5 expression in gastric cancer tissues from TCGA-STAD via GEPIA2.

**B** System diagram about the treatment time line of AGS cells.

**C** Representative images (left) and volumes (right) of the enucleated popliteal LNs in the indicated mouse groups.

**D** Ratios of metastatic and non-metastatic enucleated popliteal LNs to the total count for the indicated groups.

**E** Representative images of HE and CK-18 IHC staining of the popliteal lymph nodes for the indicated groups.

Data are expressed as the mean ± SD of biological replicate experiments. * indicates *p* < 0.05, ** indicates *p* < 0.01, *** indicates *p* < 0.001, **** indicates *p* < 0.0001, and ns indicates no significance.

**Supplementary Methods**

**MLECs isolation**

The mouse lymphatic endothelial cells (MLECs) were isolated from 615 mice (approximate age 8 weeks) as described in previous study.

**Cycloheximide (CHX) and MG132 treatment**

AGS cells (serum-starvation for 1.5 h) were pretreated with PBS or IL-10 (50 ng/ml, MCE) for 1.5 h. After stimulation with IL-1β (10 ng/ml, MCE) for 6 h, cells were further treated with CHX (100 μg/ml, MCE) and/or MG132 (20 μM, MCE) for the indicated time points.

**Ubiquitination assay**

For ubiquitination assay in AGS cells, cells were pretreated with PBS or IL-10 (50 ng/ml, MCE) for 1.5 h, and then stimulated with IL-1β (20 ng/ml, MCE) or IL-10 alone (50 ng/ml) in the presence or absence of MG132 (20 μM, MCE) for 6 h. The lysates were prepared by using Immunoprecipitation Kit (Abcam, Cambridge, MA) according to the manufacturer’s protocol and immunoprecipitated with anti-IκBζ antibody (Cell Signaling Technology, Danvers, MA) and analyzed by western blotting with anti-Ubiquitin, IκBζ and LCN2(Cell Signaling Technology).

**Supplementary Tables**

**Supplementary Table 1. Patient characteristics for RNA-seq.**

| **Patient** | **Gender** | **Age** | **TNM stage** | **Pathological grade** |
| --- | --- | --- | --- | --- |
| LN (-) | Male | 56 | T4aN0M0 | ⅡB |
| LN (-) | Male | 60 | T3N0M0 | ⅡA |
| LN (-) | Male | 78 | T4aN0M0 | ⅡB |
| LN (-) | Female | 74 | T4aN0M0 | ⅡB |
| LN (-) | Female | 65 | T3N0M0 | ⅡA |
|  |  |  |  |  |
| LN (+) | Male | 54 | T3N1M0 | ⅡB |
| LN (+) | Male | 65 | T3N2M0 | ⅢA |
| LN (+) | Male | 67 | T4aN2M0 | ⅢA |
| LN (+) | Female | 75 | T4aN3aM0 | ⅢB |
| LN (+) | Female | 67 | T4aN2M0 | ⅢA |

Note: the TNM stage were staged according to the eighth TNM staging of the International Union against Cancer (UICC)/American Joint Committee on Cancer (AJCC) system.

Abbreviation: T stage = tumor stage; T grade = tumor grade; LN = lymph node.

**Supplementary Table 2. Correlation between LCN2 and CCL5 expression and clinicopathologic characteristics of gastric cancer patients.**

| **Characteristics** | **Total (n=280)** | **LCN2 expression level** | | | **CCL5 expression level** | | | |
| --- | --- | --- | --- | --- | --- | --- | --- | --- |
|  |  | **Low** | **High** | ***P*** | | **Low** | **High** | ***P*** |
| **Gender** |  |  |  | 0.4799 | |  |  | 0.4261 |
| Male | 170 | 100 | 70 |  | | 60 | 110 |  |
| Female | 110 | 60 | 50 |  | | 44 | 66 |  |
| **Age** |  |  |  | 0.7051 | |  |  | 0.4133 |
| <60 | 60 | 33 | 27 |  | | 25 | 35 |  |
| ≥60 | 220 | 127 | 93 |  | | 79 | 141 |  |
| **T stage** |  |  |  | 0.0376 | |  |  | 0.0370 |
| <T2 | 102 | 50 | 52 |  | | 46 | 56 |  |
| ≥T2 | 178 | 110 | 68 |  | | 58 | 120 |  |
| **T grade** |  |  |  | ****** | |  |  | ***** |
| Low | 92 | 41 | 51 |  | | 42 | 50 |  |
| High | 188 | 119 | 69 |  | | 62 | 126 |  |
| **LN metastasis** |  |  |  | ******** | |  |  | ******** |
| LN (-) | 100 | 39 | 61 |  | | 57 | 43 |  |
| LN (+) | 180 | 121 | 59 |  | | 47 | 133 |  |

Abbreviation: T stage = tumor stage; T grade = tumor grade; LN = lymph node; **p* < 0.05, ***p* < 0.01, ****p* < 0.001, *****p* < 0.0001.

**Supplementary Table 3. Sequences of shRNA and siRNA used in this study.**

| **Gene name** | **Primer sequences** |
| --- | --- |
| Human-shLCN2-sense | 5'-GAGCUGACUUCGGAACUAA-3' |
| Human-shLCN2-antisense | 5'-TTAGTTCCGAAGTCAGCTC-3' |
| Human-shANXA1-sense | 5'-AUUCUAUCAGAAGAUGUAUTT-3' |
| Human-shANXA1-antisense | 5'-AUACAUCUUCUGAUAGAAUTT-3' |
| Human-shNFKBIZ-sense | 5'-AGCAAAUCUGGAACUCAUUTT-3' |
| Human-shNFKBIZ-antisense | 5'-AAUGAGUUCCAGAUUUGCUTT-3' |
| Mouse-shLCN2#1-sense | 5'-GCTACTGGATCAGAACATTTG-3' |
| Mouse-shLCN2#1-antisense | 5'-CAAATGTTCTGATCCAGTAGC-3' |
| Mouse-shLCN2#2-sense | 5'-CCAATGCATTGACAACTGAAT-3' |
| Mouse-shLCN2#2-antisense | 5'-ATTCAGTTGTCAATGCATTGG-3' |
| Mouse-shCCL5-sense | 5'-GGAGCUUACUGGCAAACAU-3' |
| Mouse-shCCL5-antisense | 5'-ATGTTTGCCAGTAAGCTCC-3' |

**Supplementary Table 4. Sequences of primers used in this study.**

| **Primer name** | **Primer sequences** |
| --- | --- |
| Human-GAPDH-F | 5'-AGCCACATCGCTCAGACAC-3' |
| Human-GAPDH-R | 5'-GCCCAATACGACCAAATCC-3' |
| Human-LCN2-F | 5'-GAGTTACCCTGGATTAACGAGT-3' |
| Human-LCN2-R | 5'-AAGCGGATGAAGTTCTCCTTTA-3' |
| Human-CCL5-F | 5'-CCAGCAGTCGTCTTTGTCAC-3' |
| Human-CCL5-R | 5'-CTCTGGGTTGGCACACACTT-3' |
| Human-IL-10-F | 5'-ACCTTATTGTACCTCTCTTAT-3' |
| Human-IL-10-R | 5'-GGGCTTCTTTCTAAATCG-3' |
| Human-NFKBIZ-F | 5'-GCACATCCGAAGTCATAAAC-3' |
| Human-NFKBIZ-R | 5'-CAGGTCCATCAGACAACGA-3' |
| Human-VEGFC-F | 5'-GAGGAGCAGTTACGGTCTGTG-3' |
| Human-VEGFC-R | 5'-TCCTTTCCTTAGCTGACACTTGT-3' |
| Human-CD86-F | 5'-GTCAGGGGTGGTTTATTGCA-3' |
| Human-CD86-R | 5'-AGTGAGGAACAAGCCAGAGC-3' |
| Human-CD206-F | 5'-GACGTGGCTGTGGATAAATAAC-3' |
| Human-CD206-R | 5'-CAGAAGACGCATGTAAAGCTAC-3' |
| Human-CD163-F | 5'-ATCAACCCTGCATCTTTAGACA-3' |
| Human-CD163-R | 5'-CTTGTTGTCACATGTGATCCAG-3' |
| Human-TNF-α-F | 5'-TCGGGCCAATGCCCTCCTGGCCAA-3' |
| Human-TNF-α-R | 5'-GTAGACCTGCCCAGACTCGGCAAA-3' |
| Human-TNF-β-F | 5'-CTAATGGTGGAAACCCACAACG-3' |
| Human-TNF-β-R | 5'-TATCGCCAGGAATTGTTGCTG-3' |
| Human-CXCL1-F | 5'-CTTGCCTCAATCCTGCATC-3' |
| Human-CXCL1-R | 5'-CCTTCTGGTCAGTTGGATTTG-3' |
| Human-CXCL3-F | 5'-CTTGTCTCAACCCCGCATC-3' |
| Human-CXCL3-R | 5'-GCTTCTTACTTCTCTCCTGTCA-3' |
| Human-CXCL6-F | 5'-TACCGAAAAGGCTGTGGATT-3' |
| Human-CXCL6-R | 5'-ATGAGAAGAGTGAGTAAGAACAACA-3' |
| Human-CXCL9-F | 5'-ATTGTGTAGGAGAGGTTGTCTG-3' |
| Human-CXCL9-R | 5'-CAGCAGTGTGAGCAGTGAT-3' |
| Human-CXCL10-F | 5'-AAACCAGAGGGGAGCAAAATCG-3' |
| Human-CXCL10-R | 5'-TGTAGGGAAGTGATGGGAGAGG-3' |
| Human-CCL2-F | 5'-CAAGCAGAAGTGGGTTCAGG-3' |
| Human-CCL2-R | 5'-GGGTTGTGGAGTGAGTGTTCA-3' |
| Human-CCL4-F | 5'-AGACACATCTCCTCCATACTCA-3' |
| Human-CCL4-R | 5'-CACCTAATACAATAACACGGCAC-3' |
| Human-IFN-γ-F | 5'-ACAGGGAAGCGAAAAAGGAGT-3' |
| Human-IFN-γ-R | 5'-GCAGGCAGGACAACCATTAC-3' |
| Human-IL-1β-F | 5'-GCCAGTGAAATGATGGCTTATT-3' |
| Human- IL-1β-R | 5'-AGGAGCACTTCATCTGTTTAGG-3' |
| Human-IL-4-F | 5'-TGAACAGCCTCACAGAGCAG-3' |
| Human- IL-4-R | 5'-GGCAGCGAGTGTCCTTCT-3' |
| Human-IL-6-F | 5'-ACCCCCAGGAGAAGATTCCA-3' |
| Human- IL-6-R | 5'-GATGCCGTCGAGGATGTACC-3' |
| Human-IL-11-F | 5'-AGCGGACAGGGAAGGGTT-3' |
| Human- IL-11-R | 5'-TCAGCACGACCAGGACCA-3' |
| Human-IL-12-F | 5'-ACTCCCAAAACCTGCTGAGG-3' |
| Human- IL-12-R | 5'-GGTAAACAGGCCTCCACTGT-3' |
| Human-IL-13-F | 5'-AGGGAAGAGCAGGAAAAGGC-3' |
| Human- IL-13-R | 5'-TGTAGAGGGAGGCACAGGG-3' |
| Human-IL-22-F | 5'-GCTTGGAGAGAGTGGAGAGAT-3' |
| Human- IL-22-R | 5'-AGCAGGGAAAGGGGGTTAGT-3' |
| Human-IL-24-F | 5'-TTGGTGAATGGTGAAGGTGC-3' |
| Human- IL-24-R | 5'-TGTCTTGTCCCTCTGGTCCT-3' |
| Mouse-GAPDH-F | 5'-ACCCAGAGGACTGTGGATGG-3' |
| Mouse-GAPDH-R | 5'-TCAGCTCTGGGATGACCTTG-3' |
| Mouse-LCN2-F | 5'-TGGCCCTGAGTGTCATGTG-3' |
| Mouse-LCN2-R | 5'-CTCTTGTAGCTCATAGATGGTGC-3' |
| Mouse-CCL5-F | 5'-CTCTGCCGCGGGTACCATGA-3' |
| Mouse-CCL5-R | 5'-TCCTTCGAGTGACAAACACGACTGC-3' |
| Mouse-IL-10-F | 5'-ATGCAGGACTTTAAGGGTTACTTG-3' |
| Mouse-IL-10-R | 5'-AGACACCTTGGTCTTGGAGCTTA-3' |
| Mouse-CD206-F | 5'-TTCTTCGATTTGGGTCTCCTTG-3' |
| Mouse-CD206-R | 5'-GTGCAGCTCTTGTCGGTGAA-3' |
| Mouse-CD163-F | 5'-GTGGACTCTGAAGCGACGACA-3' |
| Mouse-CD163-R | 5'-TCCGCCTTTGAATCCATCTC-3' |
| Mouse-TNF-α-F | 5'-CCAAACCAGCCTGACAACTT-3' |
| Mouse-TNF-α-R | 5'-TCTAGCATGCTCCACCACTG-3' |
| Mouse-TNF-β-F | 5'-CCACCTGCAAGACCATCGAC-3' |
| Mouse-TNF-β-R | 5'-CTGGCGAGCCTTAGTTTGGAC-3' |

**Supplementary Table 5. Antibodies used in this study for WB, Co-IP, IHC, IF, and Flow cytometry.**

| **Antibody** | **Application** | **Dilution** | **Supplier** | **Catalog number** |
| --- | --- | --- | --- | --- |
| Rabbit IgG | WB | 1:5000 | CST | #7074 |
| Mouse IgG | WB | 1:5000 | CST | #7076 |
| Rabbit IgG | Co-IP | 1:2000 | CST | #2729 |
| Secondary antibody | IHC | / | Servicebio | G1210-2-A |
| h-GAPDH | WB | 1:5000 | Proteintech | Cat No.60004-1-Ig |
| h-LCN2 | WB/IHC/IF/Co-IP | 1:1000/1:200/1:150/1:50 | Proteintech | Cat No.26991-1-AP |
| h/m-CCL5 | WB/IHC | 1:1000/1:200 | Abcam | ab322195 |
| h-CD68 | IF | 1:100 | Abcam | ab283654 |
| h-CD206 | WB/ IHC/IF/ | 1:1000/1:200/1:150 | Proteintech | Cat No.18704-1-AP |
| h-CD86 | IHC | 1:200 | Abcam | ab317266 |
| Flag | Co-IP | 1:500 | CST | #14793 |
| p65 | WB/IF | 1:1000/1:500 | CST | #8242 |
| p-p65 | WB | 1:1000 | CST | #3033 |
| Histone | WB | 1:1000 | Abcam | ab201456 |
| p-IKKα/β | WB | 1:1000 | CST | #2697 |
| IKKα | WB | 1:1000 | CST | #11930 |
| IKKβ | WB | 1:1000 | CST | #8943 |
| p-IκBα | WB | 1:1000 | CST | #2859 |
| IκBα | WB | 1:1000 | CST | #4814 |
| ANXA1 | WB/Co-IP | 1:1000/1:200 | CST | #32934 |
| NEMO | WB/Co-IP | 1:1000/1:200 | Proteintech | Cat No.18474-1-AP |
| K63-Ub | WB | 1:1000 | CST | #12930 |
| M1-Ub | WB | 1:500 | Millipore | MABS451 |
| Sharpin | WB | 1:1000 | CST | #12541 |
| HOIL-1 | WB | 1:1000 | Abcam | ab108479 |
| HOIP | WB | 1:1000 | CST | #99633 |
| h-LYVE-1 | IHC | 1:250 | Angiobio | 11-032 |
| h-VEGFC | WB/IHC | 1:1000/1:200 | Abcam | ab9546 |
| h-IL-10 | WB | 1:1000 | Abcam | ab34843 |
| p-GSK3β | WB | 1:1000 | CST | #9323 |
| GSK3β | WB | 1:1000 | CST | #12456 |
| p-AKT | WB | 1:1000 | CST | #4060 |
| AKT | WB | 1:1000 | CST | #4685 |
| IκBζ | WB/Co-IP | 1:1000/1:150 | CST | #9244 |
| Ubiquitin | WB | 1:1000 | CST | #3936 |
| CK18 | IHC | 1:250 | Proteintech | Cat No.10830-1-AP |
| m-LCN2 | WB/IHC | 1:1000/1:200 | Abcam | ab216462 |
| m-LYVE-1 | IHC | 1:250 | Angiobio | 11-034 |
| h-CD45-FITC | Flow Cyt | / | Biolegend | 982316 |
| h-CD68-APC | Flow Cyt | / | Biolegend | 333810 |
| h-CD206-PE | Flow Cyt | / | Biolegend | 321106 |
| h-HLA-DR-FITC | Flow Cyt | / | Biolegend | 980402 |
| Live-APC700 | Flow Cyt | / | BD | 564997 |
| CD16/CD32 | Flow Cyt |  | Biolegend |  |
| m-CD45-FITC | Flow Cyt | / | Biolegend | 103108 |
| m-F4/80-APC | Flow Cyt | / | Biolegend | 123116 |
| m-CD206-PE | Flow Cyt | / | Biolegend | 141706 |
| m-CD86-PECY7 | Flow Cyt | / | Biolegend | 105014 |
| m-CD3-FITC | Flow Cyt | / | Biolegend | 100204 |
| m-CD4-APC | Flow Cyt | / | Biolegend | 100412 |
| m-CD8a-PE | Flow Cyt | / | Biolegend | 100708 |

Abbreviations: WB, western blotting; IHC, Immunohistochemistry; Co-IP, Co-Immunoprecipitation; IF, Immunofluorescence. Flow Cyt, Flow cytometry.

**Supplementary Table 6.** **Expression of Forty-six total cytokines and chemokines in conditioned medium of GC cells with LCN2 overexpression and silencing**

| **Cytokine** | **Con (pg/ml)** | | | | **P-value** | | **Con (pg/ml)** | | | | **P-value** |
| --- | --- | --- | --- | --- | --- | --- | --- | --- | --- | --- | --- |
|  | **Vector** | **LCN2** | | |  |  | **shNC** | **shLCN2** | | |  |
| CCL2 | 140.254 | | 130.235 | 0.199211 | | 150.364 | | | 143.165 | 0.959900 | |
|  | 123.457 | | 120.765 |  |  | 134.764 | | | 134.253 |  |  |
|  | 142.124 | | 124.687 |  |  | 149.524 | | | 158.637 |  |  |
| CCL3 | 32.353 | | 29.457 | 0.246856 | | 25.457 | | | 43.234 | 0.085448 | |
|  | 26.564 | | 24.346 |  |  | 34.235 | | | 36.168 |  |  |
|  | 30.464 | | 26.387 |  |  | 30.534 | | | 35.252 |  |  |
| CCL4 | 89.568 | | 78.564 | 0.285613 | | 90.428 | | | 75.436 | 0.101573 | |
|  | 78.764 | | 65.231 |  |  | 85.276 | | | 69.548 |  |  |
|  | 70.678 | | 70.435 |  |  | 82.126 | | | 83.274 |  |  |
| CCL5 | 201.345 | | 67.564 | 0.000021 | | 223.634 | | | 654.234 | 0.000065 | |
|  | 190.673 | | 70.314 |  |  | 205.643 | | | 602.124 |  |  |
|  | 189.546 | | 56.784 |  |  | 243.531 | | | 589.746 |  |  |
| CCL11 | 30.455 | | 34.322 | 0.586125 | | 23.265 | | | 26.456 | 0.083053 | |
|  | 35.344 | | 36.564 |  |  | 21.321 | | | 24.123 |  |  |
|  | 29.456 | | 29.343 |  |  | 24.345 | | | 26.546 |  |  |
| CCL19 | 0 | | 0 | **—** | | 0 | | | 0 | **—** | |
|  | 0 | | 0 |  |  | 0 | | | 0 |  |  |
|  | 0 | | 0 |  |  | 0 | | | 0 |  |  |
| CCL20 | 56.493 | | 45.357 | 0.119433 | | 51.245 | | | 54.264 | 0.960967 | |
|  | 52.334 | | 48.753 |  |  | 43.456 | | | 41.264 |  |  |
|  | 49.846 | | 50.236 |  |  | 53.264 | | | 53.239 |  |  |
| CXCL1 | 123.313 | | 94.314 | 0.935768 | | 102.146 | | | 105.364 | 0.722894 | |
|  | 103.293 | | 102.764 |  |  | 121.146 | | | 129.419 |  |  |
|  | 87.721 | | 120.564 |  |  | 98.758 | | | 100.253 |  |  |
| CXCL2 | 78.223 | | 78.847 | 0.960825 | | 67.595 | | | 79.526 | 0.002517 | |
|  | 67.314 | | 54.324 |  |  | 59.876 | | | 80.435 |  |  |
|  | 56.727 | | 67.616 |  |  | 60.432 | | | 83.325 |  |  |
| CXCL10 | 1.345 | | 1.346 | 0.353964 | | 1.123 | | | 0.789 | 0.560073 | |
|  | 1.093 | | 1.976 |  |  | 0.897 | | | 1.034 |  |  |
|  | 1.987 | | 2.342 |  |  | 0.976 | | | 0.983 |  |  |
| lL-1α | 5.673 | | 5.453 | 0.950543 | | 8.453 | | | 7.568 | 0.907204 | |
|  | 6.876 | | 7.868 |  |  | 7.587 | | | 9.546 |  |  |
|  | 9.873 | | 8.785 |  |  | 8.489 | | | 7.678 |  |  |
| IL-1β | 0 | | 0 | **—** | | 0 | | | 0.342 | **—** | |
|  | 0.322 | | 0 |  |  | 0 | | | 0.145 |  |  |
|  | 0 | | 0 |  |  | 0 | | | 0 |  |  |
| IL-1ra | 0 | | 0 | **—** | | 0 | | | 0 | **—** | |
|  | 0 | | 0 |  |  | 0 | | | 0 |  |  |
|  | 0 | | 0 |  |  | 0 | | | 0 |  |  |
| IL-2 | 1.237 | | 1.112 | 0.563398 | | 1.567 | | | 2.024 | 0.036022 | |
|  | 2.103 | | 1.564 |  |  | 1.235 | | | 2.567 |  |  |
|  | 1.879 | | 1.893 |  |  | 1.764 | | | 3.021 |  |  |
| IL-3 | 0 | | 0 | **—** | | 0 | | | 0 | **—** | |
|  | 0 | | 0 |  |  | 0 | | | 0 |  |  |
|  | 0 | | 0 |  |  | 0 | | | 0 |  |  |
| IL-4 | 0 | | 0 | **—** | | 0 | | | 0 | **—** | |
|  | 0 | | 0 |  |  | 0 | | | 0 |  |  |
|  | 0 | | 0 |  |  | 0 | | | 0 |  |  |
| IL-5 | 0 | | 0 | **—** | | 0 | | | 0 | **—** | |
|  | 0 | | 0 |  |  | 0 | | | 0 |  |  |
|  | 0 | | 0 |  |  | 0 | | | 0 |  |  |
| IL-6 | 786.556 | | 766.395 | 0.706357 | | 723.124 | | | 700.345 | 0.942580 | |
|  | 667.565 | | 665.736 |  |  | 600.765 | | | 675.235 |  |  |
|  | 556.786 | | 668.784 |  |  | 598.768 | | | 560.753 |  |  |
| IL-7 | 0.231 | | 0.032 | 0.258107 | | 0.264 | | | 0.346 | 0.327978 | |
|  | 0.453 | | 0.345 |  |  | 0.123 | | | 0.675 |  |  |
|  | 0.345 | | 0.213 |  |  | 0.456 | | | 0.321 |  |  |
| IL-8 | 678.643 | | 869.765 | 0.008674 | | 612.564 | | | 497.464 | 0.004714 | |
|  | 556.435 | | 832.345 |  |  | 600.346 | | | 482.357 |  |  |
|  | 634.562 | | 786.784 |  |  | 650.457 | | | 530.345 |  |  |
| IL-9 | 0 | | 0 | **—** | | 0 | | | 0 | **—** | |
|  | 0 | | 0 |  |  | 0 | | | 0 |  |  |
|  | 0 | | 0 |  |  | 0 | | | 0 |  |  |
| IL-10 | 0 | | 0 | **—** | | 0 | | | 0 | **—** | |
|  | 0 | | 0 |  |  | 0 | | | 0 |  |  |
|  | 0 | | 0 |  |  | 0 | | | 0 |  |  |
| IL-12 p70 | 4.634 | | 3.498 | 0.872232 | | 3.215 | | | 4.324 | 0.720443 | |
|  | 6.498 | | 5.783 |  |  | 2.353 | | | 2.347 |  |  |
|  | 5.245 | | 6.547 |  |  | 3.012 | | | 2.675 |  |  |
| IL-13 | 10.231 | | 12.345 | 0.087650 | | 12.453 | | | 8.789 | 0.439484 | |
|  | 7.678 | | 11.321 |  |  | 14.324 | | | 10.234 |  |  |
|  | 9.764 | | 10.458 |  |  | 10.453 | | | 13.568 |  |  |
| IL-15 | 1.236 | | 1.123 | 0.587245 | | 2.367 | | | 1.213 | 0.005264 | |
|  | 1.654 | | 1.456 |  |  | 2.156 | | | 1.014 |  |  |
|  | 1.987 | | 1.786 |  |  | 1.978 | | | 1.456 |  |  |
| IL-17A | 2.387 | | 2.987 | 0.113751 | | 2.569 | | | 2.657 | 0.473658 | |
|  | 2.123 | | 3.435 |  |  | 2.984 | | | 2.987 |  |  |
|  | 2.678 | | 2.567 |  |  | 2.136 | | | 2.678 |  |  |
| IL-17E | 0 | | 0 | **—** | | 0 | | | 0 | **—** | |
|  | 0 | | 0 |  |  | 0 | | | 0 |  |  |
|  | 0 | | 0 |  |  | 0 | | | 0 |  |  |
| IL-33 | 4.345 | | 2.456 | 0.039360 | | 3.785 | | | 4.789 | 0.002764 | |
|  | 5.434 | | 2.235 |  |  | 3.123 | | | 4.986 |  |  |
|  | 3.453 | | 2.984 |  |  | 3.098 | | | 5.168 |  |  |
| G-CSF | 8.788 | | 9.566 | 0.915840 | | 9.646 | | | 10.235 | 0.141517 | |
|  | 10.455 | | 10.344 |  |  | 8.968 | | | 9.326 |  |  |
|  | 12.444 | | 12.222 |  |  | 9.025 | | | 9.982 |  |  |
| GM-CSF | 5.666 | | 5.455 | 0.537077 | | 7.564 | | | 10.345 | 0.001808 | |
|  | 6.567 | | 4.5666 |  |  | 7.437 | | | 9.363 |  |  |
|  | 7.677 | | 7.6777 |  |  | 6.896 | | | 9.956 |  |  |
| IFN-α2 | 1.237 | | 0.764 | 0.469388 | | 0.231 | | | 1.034 | 0.031985 | |
|  | 0.123 | | 0.784 |  |  | 0.456 | | | 1.221 |  |  |
|  | 0.341 | | 0.987 |  |  | 0.657 | | | 0.789 |  |  |
| IFN-β | 0 | | 0 | **—** | | 0 | | | 0 | **—** | |
|  | 0 | | 0 |  |  | 0 | | | 0 |  |  |
|  | 0 | | 0 |  |  | 0 | | | 0 |  |  |
| IFN-γ | 0 | | 0 | **—** | | 0 | | | 0 | **—** | |
|  | 0 | | 0 |  |  | 0 | | | 0 |  |  |
|  | 0 | | 0 |  |  | 0 | | | 0 |  |  |
| TNF-α | 0 | | 0 | **—** | | 0 | | | 0 | **—** | |
|  | 0 | | 0 |  |  | 0 | | | 0 |  |  |
|  | 0 | | 0 |  |  | 0 | | | 0 |  |  |
| TNF-β | 0 | | 0 | **—** | | 0 | | | 0 | **—** | |
|  | 0 | | 0 |  |  | 0 | | | 0 |  |  |
|  | 0 | | 0 |  |  | 0 | | | 0 |  |  |
| PD-L1 | 34.563 | | 45.675 | 0.642642 | | 37.532 | | | 39.896 | 0.961204 | |
|  | 45.535 | | 38.676 |  |  | 40.325 | | | 39.123 |  |  |
|  | 37.654 | | 39.346 |  |  | 38.786 | | | 37.456 |  |  |
| PDGF-AA | 1234.675 | | 1435.452 | 0.771031 | | 1433.231 | | | 1544.321 | 0.280925 | |
|  | 1678.567 | | 1678.567 |  |  | 1134.634 | | | 1333.453 |  |  |
|  | 1618.498 | | 1563.246 |  |  | 1322.453 | | | 1411.234 |  |  |
| PDGF-AB | 145.346 | | 123.986 | 0.843718 | | 132.432 | | | 165.567 | 0.014275 | |
|  | 123.586 | | 145.278 |  |  | 123.425 | | | 161.234 |  |  |
|  | 132.412 | | 137.678 |  |  | 143.234 | | | 154.345 |  |  |
| TGF-α | 1.236 | | 2.034 | 0.676326 | | 1.002 | | | 1.563 | 0.124811 | |
|  | 2.045 | | 1.894 |  |  | 1.453 | | | 1.421 |  |  |
|  | 1.932 | | 1.657 |  |  | 1.322 | | | 1.782 |  |  |
| TRAIL | 4.342 | | 2.323 | 0.044174 | | 4.784 | | | 6.536 | 0.002571 | |
|  | 3.498 | | 3.032 |  |  | 4.134 | | | 5.897 |  |  |
|  | 3.786 | | 3.154 |  |  | 4.246 | | | 6.215 |  |  |
| VEGF | 876.563 | | 756.753 | 0.601307 | | 893.235 | | | 865.234 | 0.941894 | |
|  | 789.656 | | 845.342 |  |  | 800.435 | | | 800.346 |  |  |
|  | 800.323 | | 800.789 |  |  | 823.125 | | | 843.346 |  |  |
| EGF | 0 | | 0 | **—** | | 0 | | | 0 | **—** | |
|  | 0 | | 0 |  |  | 0 | | | 0 |  |  |
|  | 0 | | 0 |  |  | 0 | | | 0 |  |  |
| FGF basic | 5.455 | | 6.544 | 0.707190 | | 3.246 | | | 6.564 | 0.015332 | |
|  | 2.333 | | 3.237 |  |  | 4.342 | | | 5.458 |  |  |
|  | 2.345 | | 2.345 |  |  | 3.467 | | | 5.323 |  |  |
| Flt-3 Ligand | 45.344 | | 32.333 | 0.010557 | | 47.458 | | | 32.542 | 0.005365 | |
|  | 56.566 | | 31.222 |  |  | 42.125 | | | 31.425 |  |  |
|  | 45.444 | | 25.459 |  |  | 48.589 | | | 35.568 |  |  |
| CD40 | 0 | | 2.455 | **—** | | 0 | | | 0.435 | **—** | |
|  | 2.233 | | 1.5566 |  |  | 0.476 | | | 0.378 |  |  |
|  | 1.451 | | 0 |  |  | 0.257 | | | 0 |  |  |
| Granzyme B | 0 | | 1.236 | **—** | | 0 | | | 0.324 | **—** | |
|  | 0.541 | | 0.001 |  |  | 0 | | | 0.312 |  |  |
|  | 0.762 | | 0.452 |  |  | 0.436 | | | 0 |  |  |
